# Role of Oxidative Stress in Ocular Diseases Associated with Retinal Ganglion Cells Degeneration

**DOI:** 10.3390/antiox10121948

**Published:** 2021-12-05

**Authors:** Eugene Yu-Chuan Kang, Pei-Kang Liu, Yao-Tseng Wen, Peter M. J. Quinn, Sarah R. Levi, Nan-Kai Wang, Rong-Kung Tsai

**Affiliations:** 1 Department of Ophthalmology, Linkou Chang Gung Memorial Hospital, Taoyuan 33302, Taiwan; yckang@cgmh.org.tw; 2 Graduate Institute of Clinical Medical Sciences, College of Medicine, Chang Gung University, Taoyuan 33302, Taiwan; 3 Department of Ophthalmology, Kaohsiung Medical University Hospital, Kaohsiung 80424, Taiwan; 990288@gap.kmu.edu.tw; 4 School of Medicine, College of Medicine, Kaohsiung Medical University, Kaohsiung 80424, Taiwan; 5 Institute of Biomedical Sciences, National Sun Yat-sen University, Kaohsiung 80424, Taiwan; 6 Edward S. Harkness Eye Institute, Department of Ophthalmology, Columbia University Irving Medical Center, New York, NY 10032, USA; 7 Institute of Eye Research, Hualien Tzu Chi Hospital, Buddhist Tzu Chi Medical Foundation, Hualien 97403, Taiwan; ytw193@tzuchi.com.tw; 8 Jonas Children’s Vision Care, and Bernard and Shirlee Brown Glaucoma Laboratory, Columbia Stem Cell Initiative, Departments of Ophthalmology, Pathology and Cell Biology, Institute of Human Nutrition, Vagelos College of Physicians and Surgeons, Columbia University, New York, NY 10032, USA; pq2138@cumc.columbia.edu (P.M.J.Q.); srl2183@cumc.columbia.edu (S.R.L.); 9 Institute of Medical Sciences, Tzu Chi University, Hualien 97403, Taiwan

**Keywords:** retinal ganglion cell, degeneration, oxidative stress, mitochondria, glaucoma, hereditary optic atrophy, ischemic optic neuropathy, traumatic optic neuropathy, optic neuritis

## Abstract

Ocular diseases associated with retinal ganglion cell (RGC) degeneration is the most common neurodegenerative disorder that causes irreversible blindness worldwide. It is characterized by visual field defects and progressive optic nerve atrophy. The underlying pathophysiology and mechanisms of RGC degeneration in several ocular diseases remain largely unknown. RGCs are a population of central nervous system neurons, with their soma located in the retina and long axons that extend through the optic nerve to form distal terminals and connections in the brain. Because of this unique cytoarchitecture and highly compartmentalized energy demand, RGCs are highly mitochondrial-dependent for adenosine triphosphate (ATP) production. Recently, oxidative stress and mitochondrial dysfunction have been found to be the principal mechanisms in RGC degeneration as well as in other neurodegenerative disorders. Here, we review the role of oxidative stress in several ocular diseases associated with RGC degenerations, including glaucoma, hereditary optic atrophy, inflammatory optic neuritis, ischemic optic neuropathy, traumatic optic neuropathy, and drug toxicity. We also review experimental approaches using cell and animal models for research on the underlying mechanisms of RGC degeneration. Lastly, we discuss the application of antioxidants as a potential future therapy for the ocular diseases associated with RGC degenerations.

## 1. Introduction

Retinal ganglion cells (RGCs) have multiple functions including the communication between photoreceptors and the brain, processing visual signals, and controlling visual information. [1,2,3]. When rod and cone photoreceptors are activated by light stimulation, they transmit the signal first to interneurons and then to RGCs, where signal processing takes place. Then, the signals are transmitted to the central nervous system (CNS) in order to generate the original image [4]. Given RGCs’ relatively large size/long length compared to other human neural cells and their frequent transmission of visual stimulation, RGCs’ energy demand is high and requires efficient energy production with adenosine triphosphate (ATP) [5]. One reason for the high ATP requirements can be explained by the characteristic of RGCs’ axons, which remain unmyelinated within the retina. Specifically, these axons do not experience saltatory conduction, and as such, require a larger ATP supply to propagate an action potential, as compared to the energy requirements in the myelinated portion of axons after passing through lamina cribrosa [6]. During the energy production, reactive oxidative species (ROS) are generated from the electron transport chain in mitochondria. Under normal conditions, the ROS could be mediated by mitochondrial proteins and other scavengers [7]. However, homeostasis between the generation and scavenge of ROS can be broken when the electron transport chain or mitochondrial function is impaired [8]. Once the balance of ROS is broken, it can lead to oxidative stress that damages mitochondrial or nuclear chromosomes, compromising cellular function, ultimately leading to cell death.

Since the concept of oxidative stress was introduced in the recent 50 years [9], several investigations have been published regarding its pathophysiological role in various human diseases. Previous investigations have demonstrated increased oxidative stress in neurodegenerative diseases involving the brain [10,11]. In the last decade alone, the association between oxidative stress and ocular diseases has been investigated and discussed [11,12,13]. While RGC degeneration is the main pathology in several ocular diseases such as glaucoma, any cause of RGC dysfunction or degeneration can lead to impaired visual pathways, causing ocular diseases [14]. Herein, we review the latest findings and ongoing experimental studies regarding the role of oxidative stress in eye diseases, that which is related to RGC degeneration in the visual pathway. We further review the various studies investigating antioxidant therapeutics as a treatment for such RGC degenerative eye diseases.

## 2. Mitochondria and Oxidative Stress in Retinal Ganglion Cells

**Figure 1:**
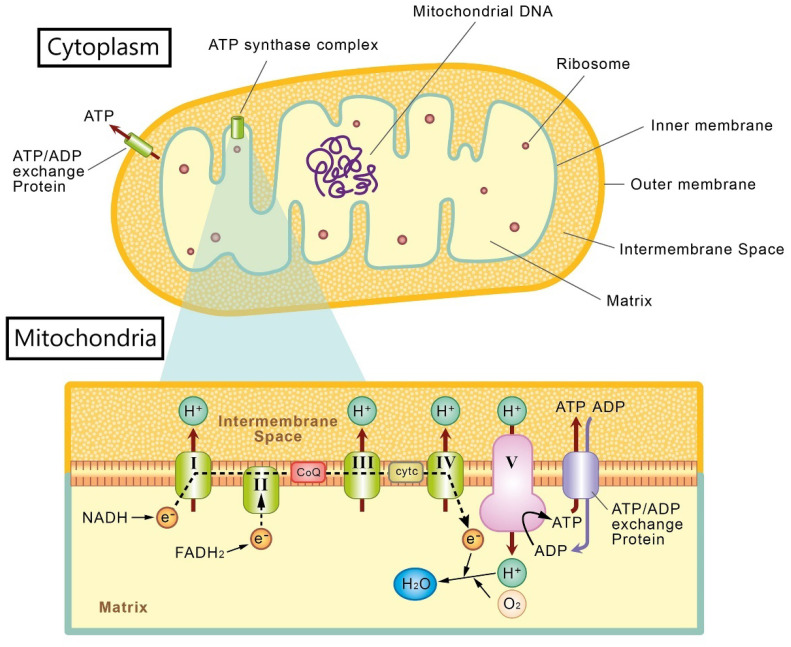
Illustration of mitochondrial structure and the electron transport chain.

The mitochondria consists of two membranes (i.e., outer and inner membranes), forming an intermembrane space between the membranes, and a separate matrix found within the inner membrane [15]. The most important function of the mitochondria is its role in energy production to maintain cellular function [16]. ATP, the major energy source of cells, is generated from the electron transport chain, a process that takes place in the inner membrane of mitochondria through oxidative phosphorylation (OXPHOS) [17]. In the electron transport chain, there are five protein complexes (I, II, III, IV, and V). From complex I to complex IV, electrons are shuttled from reduced nicotinamide adenine dinucleotide (NADH) or flavin adenine dinucleotide through ubiquinone (coenzyme Q[CoQ]) and cytochrome *c*, to the final product, hydrogen dioxide. The complexes also generate a proton gradient between intermembrane space and matrix, for the final step in ATP production. Complex V, also termed ATP synthase, uses the proton gradient to phosphorylate adenosine diphosphate (ADP) and produce ATP [15]. Then, the generated ATP is transported to the cytoplasm to supply the cellular need via the ATP/ADP exchange proteins in the inner and outer membranes (Figure 1) [18]. Mitochondria also take part in other cellular functions such as metabolism of ROS, balancing intracellular calcium levels, biosynthesis of cell structural components, and signaling for apoptosis (i.e., programmed cell death) [19]. The major signal for apoptosis is cytochrome *c,* located in the mitochondrial intermembrane space, which can be induced by cellular stress or injuries [20]. Furthermore, mitochondria are highly dynamic organelles in response to different functional states and toxic conditions [21,22]. The major dynamic characteristics of mitochondria are fusion and fission [23], which are important for mitochondria to change its morphology and maintain its functional properties [19].

Different from other organelles, mitochondria contain their own, maternally inherited, chromosomal DNA within its matrix, namely, mitochondrial DNA (mtDNA) [24]. The double-stranded and circular mtDNA is made of 16.6 k DNA base pairs and contains 37 genes that participate in energy production, as well as ribosome and transfer RNA production necessary for protein synthesis [25,26,27]. Transcription and translation of mtDNA is regulated by the non-coding region of mtDNA as well as nuclear DNA [28], and can independently undergo such processes in the mitochondrial matrix via its own ribosomes. Furthermore, mtDNA can be exchanged and mixed to further maintain and optimize mitochondrial function via mitochondrial fusion [29]. Although mtDNA encodes some mitochondrial proteins, most of the mitochondrial proteins are produced from the nuclear genome, in order to maintain normal mitochondrial functioning [22].

ROS are formed by the inclusion of free radicals with unpaired electron such as superoxide anion radical, hydroxyl radical, oxygen peroxide, singlet oxygen, and nitric oxide. In physiological condition, ROS are produced during energy production, metabolic processes, and cellular responses to infection, inflammation, or hypoxia. ROS are also generated when one is exposed to exogenous toxic factors such as radiation, smoking, toxicants, and alcohol [30,31]. In a healthy status, ROS can be depleted by several antioxidative mechanisms including enzymes (superoxide dismutase, catalase, glutathione), proteins (ferritin), radical scavengers (CoQ, uric acid), and exogenous antioxidants (vitamin A, vitamin C, vitamin E, omega-3 fatty acid) [31,32]. Once the balance between ROS production and depletion is disrupted, the accumulation of ROS generates oxidative stress that can directly damage DNA, proteins, and lipid molecules, resulting in genetic mutation, loss of cell integrity, impaired cell function, or even cell death [33,34].

Mitochondria are one of the major sources of ROS production and oxidative stress. In mitochondria, ROS are yielded from the electron transport chain during OXPHOS, with the highest ROS production activity occurring from complexes I and III [35]. In OXPHOS, the electrons leak from the mitochondrial inner membrane’s reduced oxygen molecule and generate ROS [36]. The proximity of ROS generation in mitochondria also makes mtDNA vulnerable to oxidative stress [37]. Furthermore, the elevation of oxidative stress increases mitochondrial cytochrome *c* release, which initiates the apoptosis cascade, ultimately leading to cell death [38]. Therefore, mitochondria are not only a major source of ROS, but also they are particularly susceptible to ROS-induced damage.

**Figure 2:**
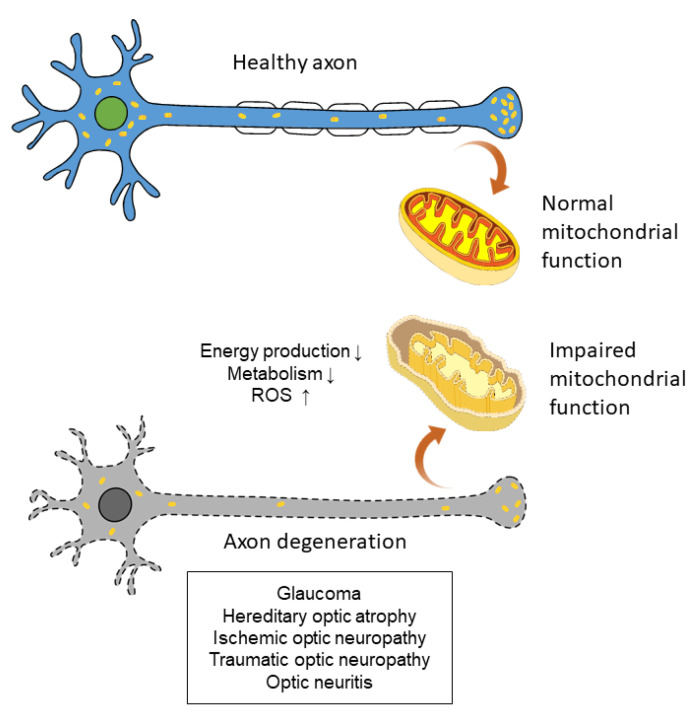
Impaired mitochondrial function can lead to axon degeneration and subsequent eye diseases.

As a site of active photoreaction and direct exposure to light, which is known to induce ROS production [39], retinal cell function is closely related to ROS homeostasis and oxidative stress [11]. In retina, mobile zinc and nitric oxide, which are closely related to oxidative stress, have been reported to be increased after optic nerve injuries [40]. Furthermore, the high energy demand of RGCs—especially of unmyelinated RGC axons within the retina—results in a highly active energy production state in mitochondria. When mitochondrial function is impaired, accumulation of ROS leads to elevated oxidative stress and, subsequently, significant mitochondrial physiology damage [41]. This vicious cycle makes the RGCs highly susceptible to ROS and oxidative stress [42]. Therefore, RGC axon degeneration has a closed association between mitochondrial function and oxidative stress (Figure 2). Recent studies also showed that the death or survival of RGCs, as well as their ability to regenerate axons, are also influenced by the complex circuitry of the retina and adjacent cells such as amacrine cells, oligodendrocytes, and bipolar cells [40,43,44]. Many studies have reported the possible pathophysiology of oxidative stress in RGCs and relevant eye diseases, as we have reviewed below.

## 3. Experimental Investigations of Oxidative Stress in Ocular Diseases with Retinal Ganglion Cell Degeneration

### 3.1. Glaucoma

Glaucoma is a degenerative disease resulting in the loss of RGCs and subsequent damage to the optic nerve [45]. Specifically, RGC apoptosis in glaucoma is caused by several pathophysiological factors, including elevation of intraocular pressure (IOP), vascular insufficiency, and oxidative stress [46,47,48,49]. Oxidative stress in glaucoma was first proposed in 1981 [50], and the association of oxidative stress and RGC loss in glaucoma via different pathways has been investigated and reviewed extensively [12,13,51]. According to the previous study in glaucoma patients, intraocular oxidative stress results in a direct damage to RGCs and indirectly leads to RGC apoptosis through activation of caspases [52]. Furthermore, intraocular oxidative stress can induce IOP elevation and is correlated to the degeneration of trabecular meshwork, which regulates aqueous humor outflow and IOP in patients with primary open-angle glaucoma (POAG) [53]. The impaired autoregulation of blood flow in the optic nerve is also associated with intraocular oxidative stress [54,55]. Regarding systemic oxidative stress, a decreased serum level of glutathione (GSH), an intrinsic antioxidant, was reported as a risk factor in patients with glaucoma [56,57]. Urinary 8-hydroxy-2′-deoxyguanosine, a marker of systemic oxidative damage, was higher in patients with normal tension glaucoma (NTG) [58]. In addition, increased systemic biomarkers of oxidative stress were associated with decreased ocular blood flow in patients with NTG [59].

In the pathogenesis of glaucoma, the RGC loss by apoptosis was observed in experimental and human glaucoma [60,61]. Mitochondria has a major role in the regulation of the apoptotic process related to RGC degeneration, and dysfunction of mitochondria leads to impaired ATP production, blockage of axonal transduction, and ROS accumulation in RGCs [52,62,63,64,65]. In addition to mitochondria, the endoplasmic reticulum (ER) also plays a crucial role in glaucoma-related RGC loss [52,66,67]. The ER mediates protein folding acts as a cellular sensor responding to ROS. Impaired ER function results in accumulation of ROS and leads to oxidative stress, also termed ER stress [11,67,68]. Previous studies of glaucoma animal model have already investigated the characteristics of oxidative stress in glaucoma (Table 1).

### 3.2. Hereditary Optic Atrophy

Mitochondrial mutational disease was first identified in Leber’s hereditary optic neuropathy (LHON) in 1988 by Wallace et al. [77]. LHON is caused by a mtDNA mutation involving insufficient cellular energy production in RGCs, being one of the most common maternally inherited mitochondrial diseases with a prevalence of approximately 1 in 50,000 [78,79]. The LHON mutations in mtDNA encoding NADH dehydrogenase (ND subunit 1 [MT-ND1] 3460G>A, MT-ND4 11778G>A, and MT-ND6 14484T>C) affect the critical subunits of NADH—ubiquinone oxidoreductase of OXPHOS complex I [80]—leading to the degeneration of RGCs and subsequent bilateral loss of central vision [77,78,81,82,83]. Complex I of the electron transport chain has been known as a major site for ROS production in the inner membrane of mitochondria [84]. The mtDNA mutations affect the physiological function of complex I, and thus lead to altered mitochondrial function, generation of superoxide radicals, accumulation of ROS, respiratory insufficiency, and reduced synthesis of ATP [85,86,87,88]. Furthermore, this energy depletion and escalating ROS in mitochondria may induce degeneration and apoptosis of RGCs in LHON [89,90,91]. There were experimental reports demonstrating the pivotal role of oxidative stress in the pathophysiology of LHON (Table 2). In addition to experimental studies, Rovcanin et al. has reported an increased level of oxidative stress in plasma in LHON patients [92].

Sharing a similar clinical presentation with LHON, autosomal-dominant optic atrophy (DOA), which is caused by the *OPA1* gene mutation [93], was also related to impaired mitochondrial function. DOA is the most common inherited optic nerve disease with the prevalence of approximately 1 in 35,000 [94,95]. Unlike the mtDNA leading to LHON, *OPA1* mutations in DOA are located in nuclear DNA [96]. OPA1 proteins on the mitochondrial inner membrane involve the control of mitochondrial remodeling and fusion [97]. Sun et al. found that a deficiency of OPA1 could lead to alteration of mitochondrial morphology and impaired respiratory function in RGCs [98]. Specifically, the *OPA1* mutation leads to impaired fusion of dysfunctional mitochondria and subsequent ROS accumulation [96]. In addition, decreased expression and protein level of superoxide dismutases-2, an antioxidant enzyme, was also found [99].

**Table 2:** Role of oxidative stress in hereditary optic atrophy.

Publication	Study Model	Results
Wong et al., 2002 [100]	cybrid cells	Differentiation of LHON cells to neuronal forms resulted in significant increases in ROS production.
Beretta et al., 2004 [101]	cybrid cells	Impaired activity of the EAAT1 glutamate transporter and enhanced ROS production in LHON cells.
Danielson et al., 2005 [102]	cybrid cells	Increased levels of sorbitol, which has been linked to oxidative stress, were noted in LHON cells.
Floreani et al., 2005 [103]	cybrid cells	Decreased antioxidant defenses and increased oxidative stress in LHON cells.
Nguyen et al., 2011 [99]	mice	OPA1 gene mutations decreased antioxidant enzyme gene and protein expression.
Lin et al., 2012 [104]	mice	Increased ROS production in both mitochondrial and synaptosome analysis in the LHON mice model.

### 3.3. Ischemic Optic Neuropathy

Non-arteritic anterior ischemic optic neuropathy (NAION) is the most common acute optic neuropathy in individuals older than 50 years of age [105]. It is caused by infarction of the short posterior ciliary arteries, followed by optic nerve head ischemia. Axonal edema and a compartment syndrome in an already crowded optic disc leads to a vicious cycle [106]. The loss of RGCs takes place as a result of the ischemic insult of the optic nerve. There have been limited experimental studies focusing on oxidative stress in NAION. Kim et al. has reported an increased expression of oxidative protein in rabbit optic nerve head after ischemic optic neuropathy induced by endothelin-1 delivered to anterior optic nerve [107].

Although ROS play critical roles in many biological processes, such as adaptation to stress, cell signaling and gene transcription regulation, differentiation, immune response, homeostasis, apoptosis, and autophagy, in normal physiological circumstances, the production of ROS far surpasses the physiological amounts, which cause destructive effects in ischemic tissues [108,109]. In the human CNS ischemic insults, ROS overproduction is a remarkable characteristic and an important mediator of ischemic damage [110]. One in vivo study in particular demonstrated that a constant escalation of ROS production will take place, following the occlusion of middle cerebral artery in rat. Following infarction, reperfusion resulted in ischemia/reperfusion injury, which induced a second peak of ROS generation fueled by the restoration of oxygen to the ischemic tissue [111,112]. The sources of ROS during infarction include mitochondrial electron transport chain, xanthine oxidase, and cyclooxygenases metabolism of arachidonic acid [113,114,115,116]. The accumulation of ROS will ultimately lead to destruction of intracellular structures, mitochondrial dysfunction, impairment of the DNA repair system, induction of apoptosis, disruption of blood–optic nerve barrier, increased neurotoxin production, and upregulation of immune-mediated neuronal injuries [117,118,119]. Additionally, a large proportion of axonal mitochondria are stationary. However, the axonal transport of mitochondria is crucial for neuronal survival and function. The ischemia/reperfusion injury in neurons will induce somatic autophagy and elimination of axonal mitochondria, which impede the delivery of energy throughout axons and elicit axonal damage [120,121,122,123].

### 3.4. Traumatic Optic Neuropathy

Blunt injury of optic nerve can induce ROS accumulation, which leads to increased production of retinal superoxide, decreased activity of superoxide dismutase 2, and activation of the inflammasome. ROS is a major contributor to secondary axon degeneration after neurological trauma to the visual system [124]. Traumatic optic neuropathy (TON) is caused by optic nerve injury after trauma, usually CNS trauma [125,126]. The pathophysiology mechanism included not only direct traumatic injury but also ischemia related to vascular insults [125]. Furthermore, post-traumatic inflammation, demyelination, or mass effects from surrounding swollen tissue may also lead to RGC loss in TON [127,128]. There have been several models established for investigating pathogenesis and potential managements of TON [129]. Similar to the mechanism behind CNS injury, RGC apoptosis following injury could be caused by the loss of neurotrophin, as well as oxidative damage from inflammatory cells [12].

Previous studies investigating the role of oxidative stress in TON have revealed a number of underlying roles [130]. The TON models from those investigations are listed in Table 3. Evidence of oxidative stress in TON was found in direct elevation of ROS and indirect increases of the biomarkers, including calcium reflux and oxidative proteins expression after the injury [131,132,133]. Mitochondrial calcium overload was found to be related to increased oxidative stress [134]. Rather than primary trauma insult, secondary degeneration of neuron cells after trauma was found in CNS injury as well as TON [131,135,136]. In secondary degeneration of TON, elevated oxidative stress was found late after the injury [137,138], and a change of mitochondrial structure and physiology were identified [139].

### 3.5. Optic Neuritis

Inflammation in the optic nerve affects neural function and impairs visual transmission to the CNS [142]. Optic neuritis (ON), a condition characterize by inflammation of the optic nerve, is commonly idiopathic but may be associated with systemic diseases including multiple sclerosis (MS) and neuromyelitis optica (NMO) [143]. In ON, the inflammation leads to the breakdown of the blood–optic nerve barrier, demyelination, and death of RGCs [144]. Similar to the mechanism behind TON, the RGC loss can be primary as in the demyelination of MS (a disseminated demyelinating disease), or secondary to the primary inflammation [142,145]. The pathophysiology of inflammatory disease is closely related to oxidative stress, and either inflammation or ROS accumulation is able to induce the other [146]. A clinical study found elevated serum gamma glutamyl-transferase, an early marker of oxidative stress, in patients with MS and NMO [147]. Although anti-inflammatory therapy such as steroid treatment is frequently used for treating ON, oral lipoic acid has been proposed as a potential antioxidative treatment for ON [148].

The role of oxidative stress in ON has been suggested since the 1980s [149], with countless studies implementing the experimental autoimmune encephalomyelitis model for ON studies. Previous experimental studies demonstrating the role of oxidative stress in ON is listed in Table 4. Target oxidative stress inhibitors such as apoptosis signal-regulating kinase-1 inhibitor were investigated and indirectly uncovered the major role of oxidative stress in the pathogenesis of ON [150]: impaired mitochondrial function [151].

## 4. Experimental Models of Retinal Ganglion Cell Degeneration to Study Oxidative Stress

As several cell and animal models have been used to investigate the role of oxidative stress in RGC degeneration, here, we review the critical experimental models used in the aforementioned investigations.

### 4.1. Glaucoma Model

Several experimental models with elevated IOP were used to recapitulate glaucoma, including hyaluronic acid/saline/silicon oil injection to the eyes, episcleral vein cauterization, ischemia reperfusion (I/R) injury model, knockouts of glutamate/aspartate transport gene, and inbred DBA/2J mice [69,70,71,72,73,74,157,158]. Injecting 25 μL of 1% hyaluronic acid, 2 μL 15-μm polystyrene microbeads, or silicon oil into rat or mice anterior chambers can induce significant elevation of IOP [69,76,157,159], and the injection can be repeated several times for prolonged IOP elevation—this produces a chronic glaucoma model. Silicon oil in the anterior chamber can be removed to reopen an aqueous outflow pathway and return IOP to a normal level [157]. To impede the outflow of the aqueous humor, obstruction of the episcleral vein, laser trabecular photocoagulation, or laser buns to limbal vessels have been applied [160,161,162]. In the episcleral vein cauterization models, sustained elevation of IOP was induced by low-temperature cauterization of three episcleral veins [163], and the IOP could reach up to 90% higher than in untreated mice for one month. Retinal I/R injury was performed using phosphate-buffered saline infusion to the anterior chamber of C57BL6/J mice to elevate IOP up to 90 mmHg for 60 min. The I/R injury model could be used for representing acute angle closure glaucoma attack [158]. Glutamate/aspartate transporter in glial cells protect neural cells from glutamate neurotoxicity, and the deficiency of the glutamate/aspartate transporter gene could lead to RGC death; this manipulation represents a normal tension glaucoma model [74]. For the best representation of congenital glaucoma model, the DBA/2J inbred mouse strain was first described in 1995 [164], and since then, the model has been used for several studies in inherited and pigmentary glaucoma [165,166,167].

### 4.2. Hereditary Optic Atrophy Model

Cybrid, or cytoplasmic hybrid, incorporates both mitochondria and mtDNA, and presents a cellular model capable of investigating mitochondrial diseases [168]. For studying LHON, Ntera 2/D1 (NT2) cybrid cell line was first created by using a neuronal precursor of a patient’s lymphoblast that contained mtDNA mutations in 11778 and 3460, the most common mtDNA mutations for LHON [100]. Other cybrids with osteosarcoma-derived LHON cell lines were later developed to contain mtDNA 11778, 3460, and 14484 mutations [101,102,103]. To gain a better understanding of the pathophysiology of hereditary optic atrophy, researchers created animal models with the equivalent mtDNA mutation (*ND* gene mutation) representing LHON [83,104,169,170], and with the murine *OPA1* gene mutation representing DOA [99,171,172]. *ND6* P25L mice carry a mutation in the *ND6* gene at 13997G>A (P25L), which is equivalent to the human *ND6* 14600G>A (P25L) mutation, thus being capable of approximating the LHON phenotype. Biochemical characteristics of *ND6* P25L mice include declined axonal number in the ON, reduction in complex I activity, decreased mitochondrial oxygen consumption, increased abnormal mitochondria, and increased ROS production. These findings suggest that oxidative stress plays a critical role in the primary pathophysiology of LHON [83,104].

### 4.3. Ischemic Optic Neuropathy Model

A rat model of anterior ischemic optic neuropathy (rAION) is a well-established animal model that recapitulates NAION. rAION can be induced by photodynamic activation of rose Bengal, which produces free radical oxygen at the optic nerve head (ONH) and establishes an environment of oxidative stress leading to ischemic optic neuropathy and subsequent RGC death [173,174,175,176]. In short, immediately after intravenous injection of 2.5 mM rose Bengal in PBS (1 mL/kg animal weight), the process consists of exposing the ONH to an argon green laser for photoactivation using a fundus lens [176,177,178].

### 4.4. Traumatic Optic Neuropathy Model

TON in human can be recapitulated in mice models [124,179,180,181]. There are two major TON animal models used in previous studies, namely, optic nerve crush (ONC) and ocular blast injury. ONC triggers axonal degeneration and subsequent RGC loss, providing a reproducible rat animal model of TON to observe RGC apoptosis and loss in a predictable manner [182,183]. The ONC animal models have been commonly adopted to study RGC apoptosis induced by oxidative stress [184,185]. In the ONC model, an incision on the temporal conjunctiva of the rat is made, followed by the detachment of the lateral rectus muscle under an operating microscope. The optic nerve is then exposed and separated with the maximal preservation of the small vessels surrounding optic nerve. The optic nerve is then clamped with a vascular clip at a distance of 2 mm posterior to the globe for 10–30 s to ensure reproducible injury in each animal. In order for the retinal vascular patency to be ascertained after the injury, the retina is checked under the surgical microscope immediately after the injury [185,186,187,188].

Ocular blast injury model was also established for TON studies [124,179,180,181,189]. The direct ocular blast damages the optic nerve while simultaneously preventing potentially confounding injury to visual pathways of the CNS [181,190]. To induce direct blast injury, researchers exposed the eyes to one or more air blasts with pressure around 15 to 30 psi for a certain duration and interval [179,181,189,190]. The rest of the body was protected from the blast wave. This injury induced a temporary elevation of IOP, followed by RGC death and axonal degeneration, which was similar throughout the length of the optic nerve. Although the lens in the mouse eye is large, the incidence of cataracts after blast injury is about 7%, which is comparable to that in the blast-exposed human eyes [181,191]. The model can also be used in the investigation of traumatic brain injury setting [189].

### 4.5. Optic Neuritis Model

The previously described ON model was studied mostly on the basis of animal models with experimental allergic encephalomyelitis (EAE) [152,156,192,193], a condition defined as inflammation in the CNS. This has also been used to model MS [194]. The encephalomyelitis was induced by sensitization of the animal to myelin antigens in combination with different adjuvants via subcutaneous injection [194,195]. To directly induce inflammation in the optic nerve, researchers have used bacterial lipopolysaccharide for a single microinjection in the rat optic nerve [196,197]. This model could represent ON without systemic or CNS inflammation.

### 4.6. Induced Pluripotent Stem Cell-Derived Retinal Ganglion Cells

Induced pluripotent stem cell (iPSC)-derived RGCs are a unique tool for investigating the role of oxidative stress caused by RGC degeneration. A plethora of studies now show the use of iPSCs for the reproducible and efficient generation of RGCs [198,199,200,201,202]. Further, studies have shown the ability of iPSC-derived RGCs to form numerous subtypes and undergo significant morphological and functional maturation when co-cultured with astrocytes [203,204]. The continued improvements in development of human iPSC-derived retinal organoids and more recently complex retina–thalamic–cortical assembloids will further fuel our understanding of the role of oxidative stress in RGC degeneration [205,206,207,208]. In particular, the development of retina–thalamic–cortical assembloids have overcome the issue of RGC loss in long-term cultures, showing increased survival compared to RGCs grown within retinal organoids [207]. In this section, we briefly highlight some of the iPSC-derived RGC disease models that have been developed using 2D and 3D culture approaches. Several teams have now made patient derived and CRISPR/Cas edited iPSC lines for the investigation of glaucoma [199,209,210], LHON [211,212,213,214], and DOA [215,216,217,218].

Several studies have modeled different types of glaucoma using patient iPSC-derived RGCs [199,209,210]. Tank binding kinase 1 (TBK1) and optineurin (OPTN) are both linked to glaucoma in addition to being key components of autophagy, with TBK1 phosphorylation of OPTN prompting selective autophagy of damaged and dysfunctional mitochondria [199,209,219]. Duplication of the *TBK1* gene is associated with NTG and leads to an increase in TBK1 expression in patient iPSC-derived RGCs. Further, Tucker et al. identified over-activation of autophagy as determined by an increase in expression of LC3-II in the patient iPSC-derived RGCs [209]. The effect of the NTG-associated E50K OPTN mutation has been modeled using patient iPSC-derived RGCs, showing increased apoptosis compared with controls. Interestingly, apoptosis, as measured levels of cleaved caspase-3, could be significantly reduced upon treatment of patient iPSC-derived RGCs with brain-derived neurotrophic factor (BDNF) and pigment epithelial-derived factor (PEDF) [199]. In a separate study, Inagaki and colleagues found that timolol, a β-adrenergic receptor (β-AR) antagonist, could decrease the levels of apoptosis and E50K mutation in the *OPTN* gene (OPTNE50K) aggregation in patient iPSC-derived RGCs by enhancing autophagic flux [220]. More recently, VanderWall et al. performed a comprehensive study on the neurodegenerative phenotypes exhibited in retinal organoid models of OPTNE50K. In this study, they both knocked-in and corrected the OPTNE50K mutation in healthy and patient iPSCs, respectively, using CRISPR/Cas9 mediated homology-directed repair (HDR) [221]. They found that at later stages of maturation OPTNE50K RGCs demonstrated neurite retraction, increased apoptosis, autophagy dysfunction, and increased excitability. Upon treatment with rapamycin, an inducer of autophagy, *OPTNE50K* retinal organoids showed decreased LC3 and levels of caspase-3, suggesting a link between autophagy and apoptotic pathways in this model [221]. An iPSC-derived RGC model of POAG from a patient with a c.412C>A mutation in the *SIX6* gene when compared to control showed a significant decrease in efficiency to generate RGCs [210]. Furthermore, Teotia et al. found that iPSC-derived RGCs that possess the *SIX6* risk allele have neurite dysfunction, increased apoptosis, a reduced expression of guidance molecules such as ROBO2, and display immature electrophysiological properties as well as abnormal calcium transients. Lastly, this model showed global dysregulation of genes involved with developmentally relevant biological processes for RGC differentiation and signaling pathways, such as mammalian target of rapamycin (mTOR) [210].

Several studies have also modeled LHON using patient iPSC-derived RGCs [211,212,213,214]. In particular, Wong et al. found that iPSC-derived RGCs from a patient with homoplasmic double mtDNA mutations m.4160T>C and m.14484T>C showed increased apoptosis and mitochondrial superoxide compared to isogenic controls. Interestingly, in the patient’s fibroblasts, they implemented cybrid technology to replace the patient’s mutant mtDNA with wild-type mtDNA from kerationcytes and subsequently generate isogenic control iPSCs from these [211]. In a study by Wu et al., they compared control iPSC-derived RGCs against RGCs derived from a LHON-affected individual and an unaffected family member who both harbored the most frequent LHON mutation, m.11778G>A [212]. Upon analysis they found that iPSC-derived RGCs from the LHON-affected individual had defective neurite outgrowth, having both less but also shorter RGC axons. The LHON-unaffected individual had comparable neurite outgrowth to the control but was found to have significant expression of the *SNCG*, which plays a role in neurofilament network integrity—this may be one contributing factor in the incomplete penetrance found for LHON [212]. Further, they found increased activation of global mitochondrial biogenesis, decreased basal respiration, and spare respiratory capacity, in addition to higher level of oxidative stress in RGCs from both LHON-affected and unaffected individuals compared to controls. Surprisingly, increased complex 1 activity was found in the LHON-unaffected but not affected individual [212]. In a follow up study, Yang and colleagues further identified differences in iPSC-derived RGCs in LHON-affected and unaffected individuals, finding both to have decreased ROS levels compared with controls. However, only the LHON-affected iPSC-derived RGCs had an increase in apoptosis [213]. Analysis of mitochondrial movement along RGC axons showed similar levels of anterograde, retrograde, and stationary movement patterns in control and LHON-unaffected lines. LHON-affected RGC axons, however, showed increased retrograde and decreased stationary mitochondrial movement patterns by comparison. Dysregulation of anterograde and retrograde proteins were found with kinesin family member 5A (KIF5A) being significantly downregulated in LHON-affected iPSC-derived RGCs compared to affected and control lines. The reduction of KIF5A in LHON-affected iPSC-derived RGCs was found to be ROS-dependent. Treatment with N-acetyl-L-cysteine (NAC), a scavenger of ROS, restored both the expression of KIF5A but also the normal mitochondrial movement patterns in LHON-affected iPSC-derived RGCs [213]. In another study examining the role of the m.11778G>A LHON mutation from the same group, Yang et al. enriched a single pool of RGCs using a modified differentiation protocol [214]. Interestingly, they found that the LHON-patient iPSC-derived optic vesicles (OVs) were smaller in size and that both OVs and enriched RGCs had lower expression of neuronal cytoskeletal marker TuJ1. Functional studies were carried out using whole-cell patch clamp, revealing electrophysiological dysfunction in the LHON-patient iPSC-derived RGCs compared with controls. Furthermore, on the basis of their previous studies, Yang et al. investigated the role of glutamate-associated AMPA receptors in LHON. A significant reduction in expression of AMPA receptor subunits, GluR1 and GluR2, and associated scaffold proteins in addition to dysregulated binding between the subunits and scaffold proteins was found in LHON-patient iPSC-derived RGCs [214].

Several teams have also begun trying to model DOA from patients with mutations in the *OPA1* gene [215,216,217,218]. Chen et al. found that *OPA1* mutation increased necrosis and apoptosis in the patient iPSCs and could not efficiently differentiate them into RGCs using their initial methodology. However, the use of neural induction medium, noggin, or β-estrogen helped promote the differentiation of the patient iPSCs to RGCs [215]. Recently the Cheetham group established DOA-iPSCs from dermal fibroblasts of a patient harboring the c.1334G>A: p.R445H mutation. Further, they generated an isogenic control through CRISPR/Cas9-mediated HDR. Correction of *OPA1* led to restoration of mitochondrial homeostasis, including mtDNA stability and cellular bioenergetics [218]. Together, these initial studies set a foundation for more in-depth analysis in *OPA1* patient iPSC-derived RGCs.

In summary, progress to improve the in vitro recapitulation of in vivo RGCs (via iPSC-derived retinal organoids) is creating a paradigm shift towards establishing an effective translational model of RGC degeneration and disease. Together, this will allow us to interrogate disease mechanisms of RGC degeneration in addition to facilitating the screening of therapeutics. Additionally, iPSC-derived RGCs are a potential treatment via cell therapy.

## 5. Potential Antioxidant Therapy for Retinal Ganglion Cell Degenerations

Given the possible role of oxidative stress in RGC degeneration, we have begun to see an increasing interest in antioxidative treatments. In this section, we review potential therapies involving the oxidative stress pathway, as a potential for treating the target eye diseases. A full list of each experimental antioxidant therapeutic is shown in Table 5.

### 5.1. Antioxidants in Glaucoma

In some animal studies of glaucoma, antioxidants including CoQ10, N-acetyl cysteine, α-lipoic acid, vitamin B3 (nicotinamide), and edaravone showed potential protection from RGC loss [222,223,224,225,226,228]. CoQ10 is the most common form of CoQ in humans, and its protective effect is believed to be a result of its role in electron stability within the mitochondria, as well as in reducing mitochondrial membrane depolarization and free radical scavenging [247]. Furthermore, decreased oxidative stress-related markers were found in a high IOP model receiving treatment of brimonidine, N-acetyl cysteine, α-lipoic acid, and edaravone [224,225,228]. Brimonidine has been widely used for glaucoma patients. In addition to lowering IOP, brimonidine has also been shown to have a neuroprotective effect on RGCs [248], possibly due to its antioxidative properties [150]. Similarly, a Rho kinase inhibitor was reported to lower IOP, serve as an antioxidant, and ultimately provide neuroprotective effects against glaucoma [227,249]. One such Rho kinase inhibitor, Netarsudil, has already been approved for glaucoma treatment [250].

### 5.2. Antioxidants in Hereditary Optic Atrophy

The pathophysiology of LHON and DOA has been linked to oxidative stress due to its direct pathogenesis in the mitochondria [80,91,104]. As such, several antioxidant treatments have been investigated for reducing oxidative stress in LHON and DOA [251,252]. Although most of the treatments are still under investigation in experimental studies, idebenone and EPI-743, synthetic analogues of CoQ10, have shown promising therapeutic benefits and have recently been approved for patients afflicted by LHON [231,253]. Off-label use of idebenone in DOA patients also demonstrated a potential therapeutic benefit on vision recovery [235,236].

### 5.3. Antioxidants in Ischemic Optic Neuropathy

Although the experimental studies are limited to investigating the pathophysiology of oxidative stress in NAION, some studies using antioxidants to treat NAION showed prolonged RGC survival [175,237,238]. In addition to glaucoma, brimonidine and Rho kinase inhibitors demonstrated antioxidative function and neuroprotective properties in ischemic optic neuropathy [237,238], and fasudil, one specific Rho kinase inhibitor, has been investigated in human subjects [239].

### 5.4. Antioxidants in Traumatic Optic Neuropathy

Previous studies found that antioxidants can reduce the loss of optic nerve axons, protect optic nerve projection and visual function, and prevent inflammation pathway activation in an ocular blast injury model. Blocking the accumulation of ROS and the activation of the inflammasome pathway may therefore serve as a potential post-injury intervention [124,179,180]. In a TON model, herbal extracts including ginkgo biloba and *Lithospermum erythrorhizon* were reported to have protective effects against oxidative stress [242,243]. Because calcium overload was involved in the second degeneration after TON, a calcium channel blocker has also demonstrated an antioxidative effect with protective effects on RGCs from secondary death [240]. In addition to antioxidative agents, hyperbaric oxygen (HBO) has also been reported to reduce RGC apoptosis and oxidative stress in animal models of TON [244]. However, prolonged HBO treatment may increase the serum level of ROS in the long term [254]. Moving forward, further investigation is needed to fully understand this resulting oxidative stress in treating TON with HBO.

### 5.5. Antioxidants in Optic Neuritis

Since inflammation and oxidative stress both have an important role in ON, identification of a target that treats both components simultaneously holds great potential. In particular, lipoic acid, which functions as an antioxidant, was previously used for reducing CNS neuron atrophy in MS and could also reduce the inflammatory response in an ON model [192,193]. Similar to lipoic acid, melatonin and gypenosides also demonstrated antioxidant and anti-inflammatory properties and showed therapeutic effects in experimental ON models [197,245].

## 6. Conclusions

To summarize this report, we reviewed the role of oxidative stress in RGC degeneration of the optic nerve, including glaucoma, hereditary optic atrophy, ischemic optic neuropathy, TON, and ON. Previous experimental studies have demonstrated the connection between oxidative stress and such RGC degenerative diseases. As such, oxidative stress has been proven to be a major underlying cause of RGC degeneration. Additionally, experimental models used for RGC degenerations were also described here. Some human studies, in fact, showed robust evidence of successful antioxidative treatment, but further investigative studies are required to gain more evidence into the efficacy of such therapeutic treatment options.

## Figures and Tables

**Table 1:** Role of oxidative stress in glaucoma.

Publication	Study Model	Results
Moreno et al., 2004 [69]	Rat	Decreased total retinal superoxide dismutase and catalase activities in increased intraocular pressure induced by hyaluronic acid injection to the anterior chamber.
Ko et al., 2005 [70]	Rat	ROS level and activity of antioxidant enzymes increased in elevation of intraocular pressure by cauterization of 3 episcleral veins.
Tezel et al., 2005 [71]	Rat	Increased protein oxidation levels in eyes with elevated intraocular pressure by hypertonic saline injections into episcleral veins.
Harada et al., 2007 [72]	Mice	Glutamate/aspartate transporter knockout mice had decreased glutathione level and demonstrated pathological features of NTG.
Ferreira et al., 2010 [73]	Rat	ROS levels increased in elevation of intraocular pressure by cauterization of 2 episcleral veins.
Harada et al., 2010 [74]	Mice	Deficiency of apoptosis signal-regulating kinase 1, an enzyme, leads to stress-induced RGCs apoptosis, preventing optic nerve degeneration in the NTG model.
Noro et al., 2019 [75]	Common marmoset	In the aged marmosets presented with glaucoma-like characteristics, increased expression of 4-hydroxy-2-nonenal in the inner retina and blood, and decreased glutathione in blood were found.
Naguib et al., 2021 [76]	Mice	ROS levels increased up to 5 weeks following IOP elevation and inhibition of nuclear factor E2-related factor 2 (*Nrf2*) gene, which participated in antioxidation pathway, leading to earlier axon degeneration.

**Table 3:** Role of oxidative stress in TON.

Publication	Study Model	Results
Levkovitch-Verbin et al., 2000 [140]	Mice	Overexpressing superoxide dismutase, which metabolized ROS, increased RGCs survival in eyes with crush injury of the optic nerve.
Lieven et al., 2006 [141]	Rat RGCs culture	Increased intracellular superoxide levels in retinal cell culture from rat eyes underwent optic nerve crush.
Fitzgerald et al., 2010 [131]	Rat	Increased oxidative stress associated enzyme (manganese superoxide dismutase) in the TON model with optic nerve transection.
Wells et al., 2012 [132]	Rat	Increased calcium flux and oxidative stress markers, and decreased catalase activity were found in TON model with optic nerve transection.
Ahmed et al., 2013 [133]	Mice	Increased intraocular ROS levels were found in TON mice induced by giving pressure posterior to the globe.
Szymanski et al., 2013 [137] O’Hare Doig et al., 2014 [138]	Rat	Increased ROS in secondary degeneration of TON in the model with partial optic nerve transection.
Bernardo-Colón et al., 2018 [124]	Mice	Increased retinal superoxide and decreased superoxide dismutases-2 in eyes injured by over-pressure air waves.

**Table 4:** Role of oxidative stress in ON.

Publication	Study Model	Results
Guy et al., 1989 [152]	Guinea pig	Less demyelination in optic nerve was found with antioxidant enzyme catalase intraperitoneal administration in experimental allergic encephalomyelitis (EAE) model.
Guy et al., 1990 [153]	Guinea pigs	Hydrogen peroxide reaction products were found in the retrobulbar optic nerve and optic nerve head of EAE model.
Qi et al., 2007 [154,155]	Mice	Elevated ROS was found after antigenic sensitization of the EAE model. Increased RGCs loss was noted in the group with superoxide dismutases-2 suppression.
Larabee et al., 2016 [156]	Mice	More severe optic nerve inflammation and visual deficit were found in EAE model with knockout antioxidant transcription factor (nuclear factor-E2-related factor).

**Table 5:** Potential antioxidant therapy for RGCs degenerations.

Antioxidants	Disease	Study Subjects	Findings
Coenzyme Q10 [222,223]	Glaucoma	DBA/2J mice; rat with ocular hypertension by intracameral saline injection	Diet supplemented with coenzyme Q10 could reduce oxidative stress mediated RGCs apoptosis.
N-Acetyl cysteine [224]	Glaucoma	Rat with induced ocular hypertension by sodium hyaluronate intracameral injection	Reduced retinal oxidative stress marker, malondialdehyde, caused by high intraocular pressure in rats with topical brimonidine tartrate eye drop installation and intraperitoneal N-acetyl cysteine injection.
Lipoic acid [225]	Glaucoma	DBA/2J mice	Increased expression of anti-oxidative genes and proteins, and decreased RGCs loss.
Vitamin B3 (nicotinamide) [226]	Glaucoma	DBA/2J mice	Increased RGC density, RGC soma size, and intensity of mitochondrial staining. Better electrical activity in pattern electroretinogram than control mice.
Rho kinase inhibitor K-115 [227]	Glaucoma	C57BL/6 mice	Decreased production of ROS and oxidation of lipids.
Edaravone [228]	Glaucoma	Normal tension glaucoma EAAC1-deficient mice	Reduced retinal oxidative stress and RGCs death.
Minocycline [229]	LHON	Cybrid cell with mtDNA 11778 mutation in teratoma cells	Increased the survival and conserved mitochondrial membrane potential of cybrid cells with overloading oxidative stress induced by thapsigargin.
Glutathione [230]	LHON	Cybrid cell with mtDNA 11778, 3640, and 14484 mutations in osteosarcoma cells	Prolonged survival in cells with induced oxidative injury by *tert*-butyl hydroperoxide and rotenone.
EPI-743 [231]	LHON	Human	Stop progression and improve vision in patients with LHON.
Idebenone [232,233,234]	LHON	Human	Prevent visual impairment and promote visual recovery in patients with LHON.
Idebenone [235,236]	DOA	Human	Improving and stabilizing visual function in patients with DOA.
Brimonidine [237]	NAION	Mice with photosensitization above the optic nerve head	Intraperitoneal injection of brimonidine decreased RGCs cell loss and oxidative stress.
Rho kinase inhibitor E212 [238]	NAION	Rat with laser induced optic nerve ischemia	Increased retinal superoxide dismutase activity and decreased ROS production by intravitreal injection.
Fasudil [239]	NAION	Human	Improvement of visual acuity in NAION patients with intravitreal injection.
Lomerizine [240]	TON	Rat with partial optic nerve transection	The calcium channel blocker reduced manganese superoxide dismutase expression and prevented secondary RGCs death.
Galantamine [241]	TON	Mice with eye blast injury	Reduced oxidative stress markers and inflammatory response.
Vit E [124]	TON	Mice with eye blast injury	High vitamin E diet prevented RGCs loss and decreased level of oxidative stress.
Ginkgo biloba [242]	TON	Rat with optic nerve clamping and RGCs cells with exogenous oxidative stress (H_2_O_2_)	Prolonged RGCs survival and in both in vitro and in vivo studies.
Lithospermum erythrorhizon [243]	TON	Mice with optic nerve crush	Protected RGCs from oxidative stress-induced cell death and reduced ROS production.
Hyperbaric oxygen treatment [244]	TON	Mice with optic nerve crush	Reduction of RGCs loss after hyperbaric oxygen therapy.
ROS-degradable propylene sulfide [180]	TON	Mice with eye blast injury	Reduction of inflammation and oxidative stress when combining with erythropoietin-R76E.
Lipoic acid [192,193]	ON	Experimental autoimmune encephalomyelitis mice	Decreased inflammation and prevented RGCs against oxidative damages.
Melatonin [197]	ON	Rat with lipopolysaccharide injection to optic nerve	Decreased microglial reactivity, demyelination, RGCs loss, and oxidative damages.
Gypenosides [245]	ON	Rat RGCs with exogenous oxidative stress (H_2_O_2_)	Reduced ROS production and inflammatory response and prevented RGCs from oxidative induced apoptosis.
Spermidine [246]	ON	Experimental autoimmune encephalomyelitis mice	Reduced demyelination H_2_O_2_-induced RGC damage.

## References

[1] Masland R.H. (2001). The fundamental plan of the retina. Nat. Neurosci..

[2] Famiglietti E.V., Kolb H. (1976). Structural basis for ON-and OFF-center responses in retinal ganglion cells. Science.

[3] Milosavljevic N., Storchi R., Eleftheriou C.G., Colins A., Petersen R.S., Lucas R.J. (2018). Photoreceptive retinal ganglion cells control the information rate of the optic nerve. Proc. Natl. Acad. Sci. USA.

[4] D’Souza S., Lang R.A. (2020). Retinal ganglion cell interactions shape the developing mammalian visual system. Development.

[5] Yu D.Y., Cringle S.J., Balaratnasingam C., Morgan W.H., Yu P.K., Su E.N. (2013). Retinal ganglion cells: Energetics, compartmentation, axonal transport, cytoskeletons and vulnerability. Prog. Retin. Eye Res..

[6] Wang L., Dong J., Cull G., Fortune B., Cioffi G.A. (2003). Varicosities of intraretinal ganglion cell axons in human and nonhuman primates. Investig. Ophthalmol. Vis. Sci..

[7] Ahmadinejad F., Geir Møller S., Hashemzadeh-Chaleshtori M., Bidkhori G., Jami M.S. (2017). Molecular Mechanisms behind Free Radical Scavengers Function against Oxidative Stress. Antioxidants.

[8] Indo H.P., Davidson M., Yen H.C., Suenaga S., Tomita K., Nishii T., Higuchi M., Koga Y., Ozawa T., Majima H.J. (2007). Evidence of ROS generation by mitochondria in cells with impaired electron transport chain and mitochondrial DNA damage. Mitochondrion.

[9] Paniker N.V., Srivastava S.K., Beutler E. (1970). Glutathione metabolism of the red cells. Effect of glutathione reductase deficiency on the stimulation of hexose monophosphate shunt under oxidative stress. Biochim. Biophys. Acta.

[10] Kim G.H., Kim J.E., Rhie S.J., Yoon S. (2015). The Role of Oxidative Stress in Neurodegenerative Diseases. Exp. Neurobiol..

[11] Ikawa M., Okazawa H., Nakamoto Y., Yoneda M. (2020). PET Imaging for Oxidative Stress in Neurodegenerative Disorders Associated with Mitochondrial Dysfunction. Antioxidants.

[12] Nita M., Grzybowski A. (2016). The Role of the Reactive Oxygen Species and Oxidative Stress in the Pathomechanism of the Age-Related Ocular Diseases and Other Pathologies of the Anterior and Posterior Eye Segments in Adults. Oxidative Med. Cell. Longev..

[13] Chrysostomou V., Rezania F., Trounce I.A., Crowston J.G. (2013). Oxidative stress and mitochondrial dysfunction in glaucoma. Curr. Opin. Pharmacol..

[14] Khatib T.Z., Martin K.R. (2017). Protecting retinal ganglion cells. Eye.

[15] Kühlbrandt W. (2015). Structure and function of mitochondrial membrane protein complexes. BMC Biol..

[16] Serasinghe M.N., Chipuk J.E. (2017). Mitochondrial Fission in Human Diseases. Handb. Exp. Pharmacol..

[17] Smeitink J., van den Heuvel L., DiMauro S. (2001). The genetics and pathology of oxidative phosphorylation. Nat. Rev. Genet..

[18] Ruprecht J.J., King M.S., Zögg T., Aleksandrova A.A., Pardon E., Crichton P.G., Steyaert J., Kunji E.R.S. (2019). The Molecular Mechanism of Transport by the Mitochondrial ADP/ATP Carrier. Cell.

[19] Detmer S.A., Chan D.C. (2007). Functions and dysfunctions of mitochondrial dynamics. Nat. Rev. Mol. Cell Biol..

[20] Garrido C., Galluzzi L., Brunet M., Puig P.E., Didelot C., Kroemer G. (2006). Mechanisms of cytochrome c release from mitochondria. Cell Death Differ..

[21] Karbowski M., Youle R.J. (2003). Dynamics of mitochondrial morphology in healthy cells and during apoptosis. Cell Death Differ..

[22] Ito Y.A., Di Polo A. (2017). Mitochondrial dynamics, transport, and quality control: A bottleneck for retinal ganglion cell viability in optic neuropathies. Mitochondrion.

[23] Chan D.C. (2012). Fusion and fission: Interlinked processes critical for mitochondrial health. Annu. Rev. Genet..

[24] Taanman J.W. (1999). The mitochondrial genome: Structure, transcription, translation and replication. Biochim. Biophys. Acta.

[25] Barchiesi A., Vascotto C. (2019). Transcription, Processing, and Decay of Mitochondrial RNA in Health and Disease. Int. J. Mol. Sci..

[26] Chial H., Craig J. (2008). mtDNA and mitochondrial diseases. Nat. Educ..

[27] Gustafson M.A., Sullivan E.D., Copeland W.C. (2020). Consequences of compromised mitochondrial genome integrity. DNA Repair.

[28] Barshad G., Marom S., Cohen T., Mishmar D. (2018). Mitochondrial DNA Transcription and Its Regulation: An Evolutionary Perspective. Trends Genet. TIG.

[29] Sênos Demarco R., Uyemura B.S., D’Alterio C., Jones D.L. (2019). Mitochondrial fusion regulates lipid homeostasis and stem cell maintenance in the Drosophila testis. Nat. Cell Biol..

[30] Schieber M., Chandel N.S. (2014). ROS function in redox signaling and oxidative stress. Curr. Biol..

[31] Pisoschi A.M., Pop A. (2015). The role of antioxidants in the chemistry of oxidative stress: A review. Eur. J. Med. Chem..

[32] Devasagayam T.P., Tilak J.C., Boloor K.K., Sane K.S., Ghaskadbi S.S., Lele R.D. (2004). Free radicals and antioxidants in human health: Current status and future prospects. J. Assoc. Physicians India.

[33] Kohen R., Nyska A. (2002). Oxidation of biological systems: Oxidative stress phenomena, antioxidants, redox reactions, and methods for their quantification. Toxicol. Pathol..

[34] Sies H. (1997). Oxidative stress: Oxidants and antioxidants. Exp. Physiol..

[35] Cadenas E., Davies K.J. (2000). Mitochondrial free radical generation, oxidative stress, and aging. Free Radic. Biol. Med..

[36] Koopman W.J., Nijtmans L.G., Dieteren C.E., Roestenberg P., Valsecchi F., Smeitink J.A., Willems P.H. (2010). Mammalian mitochondrial complex I: Biogenesis, regulation, and reactive oxygen species generation. Antioxid. Redox Signal.

[37] Guo C., Sun L., Chen X., Zhang D. (2013). Oxidative stress, mitochondrial damage and neurodegenerative diseases. Neural Regen. Res..

[38] Youle R.J., van der Bliek A.M. (2012). Mitochondrial fission, fusion, and stress. Science.

[39] Zorov D.B., Juhaszova M., Sollott S.J. (2014). Mitochondrial reactive oxygen species (ROS) and ROS-induced ROS release. Physiol. Rev..

[40] Li Y., Andereggen L., Yuki K., Omura K., Yin Y., Gilbert H.Y., Erdogan B., Asdourian M.S., Shrock C., de Lima S. (2017). Mobile zinc increases rapidly in the retina after optic nerve injury and regulates ganglion cell survival and optic nerve regeneration. Proc. Natl. Acad. Sci. USA.

[41] Van Houten B., Woshner V., Santos J.H. (2006). Role of mitochondrial DNA in toxic responses to oxidative stress. DNA Repair.

[42] Osborne N.N., Li G.Y., Ji D., Mortiboys H.J., Jackson S. (2008). Light affects mitochondria to cause apoptosis to cultured cells: Possible relevance to ganglion cell death in certain optic neuropathies. J. Neurochem..

[43] Sergeeva E.G., Rosenberg P.A., Benowitz L.I. (2021). Non-Cell-Autonomous Regulation of Optic Nerve Regeneration by Amacrine Cells. Front Cell NeuroSci..

[44] Nakazawa T., Nakazawa C., Matsubara A., Noda K., Hisatomi T., She H., Michaud N., Hafezi-Moghadam A., Miller J.W., Benowitz L.I. (2006). Tumor necrosis factor-alpha mediates oligodendrocyte death and delayed retinal ganglion cell loss in a mouse model of glaucoma. J. Neurosci. Off. J. Soc. Neurosci..

[45] Davis B.M., Crawley L., Pahlitzsch M., Javaid F., Cordeiro M.F. (2016). Glaucoma: The retina and beyond. Acta Neuropathol..

[46] Levkovitch-Verbin H. (2015). Retinal ganglion cell apoptotic pathway in glaucoma: Initiating and downstream mechanisms. Prog. Brain Res..

[47] Daniel S., Clark A.F., McDowell C.M. (2018). Subtype-specific response of retinal ganglion cells to optic nerve crush. Cell Death Discov..

[48] You Y., Gupta V.K., Li J.C., Klistorner A., Graham S.L. (2013). Optic neuropathies: Characteristic features and mechanisms of retinal ganglion cell loss. Rev. Neurosci..

[49] Agarwal R., Gupta S.K., Agarwal P., Saxena R., Agrawal S.S. (2009). Current concepts in the pathophysiology of glaucoma. Indian J. Ophthalmol..

[50] Alvarado J., Murphy C., Polansky J., Juster R. (1981). Age-related changes in trabecular meshwork cellularity. Investig. Ophthalmol. Vis. Sci..

[51] Harada C., Noro T., Kimura A., Guo X., Namekata K., Nakano T., Harada T. (2020). Suppression of Oxidative Stress as Potential Therapeutic Approach for Normal Tension Glaucoma. Antioxidants.

[52] Tezel G. (2006). Oxidative stress in glaucomatous neurodegeneration: Mechanisms and consequences. Prog. Retin. Eye Res..

[53] Saccà S.C., Pascotto A., Camicione P., Capris P., Izzotti A. (2005). Oxidative DNA damage in the human trabecular meshwork: Clinical correlation in patients with primary open-angle glaucoma. Arch. Ophthalmol..

[54] Feilchenfeld Z., Yücel Y.H., Gupta N. (2008). Oxidative injury to blood vessels and glia of the pre-laminar optic nerve head in human glaucoma. Exp. Eye Res..

[55] Nakazawa T. (2016). Ocular Blood Flow and Influencing Factors for Glaucoma. Asia-Pac. J. Ophthalmol..

[56] Gherghel D., Griffiths H.R., Hilton E.J., Cunliffe I.A., Hosking S.L. (2005). Systemic reduction in glutathione levels occurs in patients with primary open-angle glaucoma. Investig. Ophthalmol. Vis. Sci..

[57] Gherghel D., Mroczkowska S., Qin L. (2013). Reduction in blood glutathione levels occurs similarly in patients with primary-open angle or normal tension glaucoma. Investig. Ophthalmol. Vis. Sci..

[58] Yuki K., Murat D., Kimura I., Tsubota K. (2010). Increased serum total antioxidant status and decreased urinary 8-hydroxy-2′-deoxyguanosine levels in patients with normal-tension glaucoma. Acta OphthalMol..

[59] Himori N., Kunikata H., Shiga Y., Omodaka K., Maruyama K., Takahashi H., Nakazawa T. (2016). The association between systemic oxidative stress and ocular blood flow in patients with normal-tension glaucoma. Graefe’s Arch. Clin. Exp. Ophthalmol. Albrecht Graefes Arch. Klin. Exp. Ophthalmol..

[60] Kerrigan L.A., Zack D.J., Quigley H.A., Smith S.D., Pease M.E. (1997). TUNEL-positive ganglion cells in human primary open-angle glaucoma. Arch. Ophthalmol..

[61] Quigley H.A., Nickells R.W., Kerrigan L.A., Pease M.E., Thibault D.J., Zack D.J. (1995). Retinal ganglion cell death in experimental glaucoma and after axotomy occurs by apoptosis. Investig. Ophthalmol. Vis. Sci..

[62] Joza N., Susin S.A., Daugas E., Stanford W.L., Cho S.K., Li C.Y., Sasaki T., Elia A.J., Cheng H.Y., Ravagnan L. (2001). Essential role of the mitochondrial apoptosis-inducing factor in programmed cell death. Nature.

[63] Lee S., Van Bergen N.J., Kong G.Y., Chrysostomou V., Waugh H.S., O’Neill E.C., Crowston J.G., Trounce I.A. (2011). Mitochondrial dysfunction in glaucoma and emerging bioenergetic therapies. Exp. Eye Res..

[64] Almasieh M., Wilson A.M., Morquette B., Cueva Vargas J.L., Di Polo A. (2012). The molecular basis of retinal ganglion cell death in glaucoma. Prog. Retin. Eye Res..

[65] Osborne N.N. (2010). Mitochondria: Their role in ganglion cell death and survival in primary open angle glaucoma. Exp. Eye Res..

[66] Li G.Y., Osborne N.N. (2008). Oxidative-induced apoptosis to an immortalized ganglion cell line is caspase independent but involves the activation of poly(ADP-ribose)polymerase and apoptosis-inducing factor. Brain Res..

[67] Sano R., Reed J.C. (2013). ER stress-induced cell death mechanisms. Biochim. Biophys. Acta.

[68] Zhang S.X., Sanders E., Fliesler S.J., Wang J.J. (2014). Endoplasmic reticulum stress and the unfolded protein responses in retinal degeneration. Exp. Eye Res..

[69] Moreno M.C., Campanelli J., Sande P., Sánez D.A., Keller Sarmiento M.I., Rosenstein R.E. (2004). Retinal oxidative stress induced by high intraocular pressure. Free Radic. Biol. Med..

[70] Ko M.L., Peng P.H., Ma M.C., Ritch R., Chen C.F. (2005). Dynamic changes in reactive oxygen species and antioxidant levels in retinas in experimental glaucoma. Free Radic. Biol. Med..

[71] Tezel G., Yang X., Cai J. (2005). Proteomic identification of oxidatively modified retinal proteins in a chronic pressure-induced rat model of glaucoma. Investig. Ophthalmol. Vis. Sci..

[72] Harada T., Harada C., Nakamura K., Quah H.M., Okumura A., Namekata K., Saeki T., Aihara M., Yoshida H., Mitani A. (2007). The potential role of glutamate transporters in the pathogenesis of normal tension glaucoma. J. Clin. Investig..

[73] Ferreira S.M., Lerner S.F., Brunzini R., Reides C.G., Evelson P.A., Llesuy S.F. (2010). Time course changes of oxidative stress markers in a rat experimental glaucoma model. Investig. Ophthalmol. Vis. Sci..

[74] Harada C., Namekata K., Guo X., Yoshida H., Mitamura Y., Matsumoto Y., Tanaka K., Ichijo H., Harada T. (2010). ASK1 deficiency attenuates neural cell death in GLAST-deficient mice, a model of normal tension glaucoma. Cell Death Differ..

[75] Noro T., Namekata K., Kimura A., Azuchi Y., Hashimoto N., Moriya-Ito K., Komaki Y., Lee C.Y., Okahara N., Guo X. (2019). Normal tension glaucoma-like degeneration of the visual system in aged marmosets. Sci. Rep..

[76] Naguib S., Backstrom J.R., Gil M., Calkins D.J., Rex T.S. (2021). Retinal oxidative stress activates the NRF2/ARE pathway: An early endogenous protective response to ocular hypertension. Redox Biol..

[77] Wallace D.C., Singh G., Lott M.T., Hodge J.A., Schurr T.G., Lezza A.M., Elsas L.J., Nikoskelainen E.K. (1988). Mitochondrial DNA mutation associated with Leber’s hereditary optic neuropathy. Science.

[78] Hage R., Vignal-Clermont C. (2021). Leber Hereditary Optic Neuropathy: Review of Treatment and Management. Front. Neurol..

[79] Mascialino B., Leinonen M., Meier T. (2012). Meta-analysis of the prevalence of Leber hereditary optic neuropathy mtDNA mutations in Europe. Eur. J. Ophthalmol..

[80] Bi R., Logan I., Yao Y.G. (2017). Leber Hereditary Optic Neuropathy: A Mitochondrial Disease Unique in Many Ways. Handb. Exp. Pharmacol..

[81] Huoponen K., Vilkki J., Aula P., Nikoskelainen E.K., Savontaus M.L. (1991). A new mtDNA mutation associated with Leber hereditary optic neuroretinopathy. Am. J. Hum. Genet..

[82] Johns D.R., Neufeld M.J., Park R.D. (1992). An ND-6 mitochondrial DNA mutation associated with Leber hereditary optic neuropathy. Biochem. Biophys. Res. Commun..

[83] Wallace D.C., Lott M.T. (2017). Leber Hereditary Optic Neuropathy: Exemplar of an mtDNA Disease. Handb. Exp. Pharmacol..

[84] Murphy M.P. (2009). How mitochondria produce reactive oxygen species. Biochem. J..

[85] Kudin A.P., Bimpong-Buta N.Y., Vielhaber S., Elger C.E., Kunz W.S. (2004). Characterization of superoxide-producing sites in isolated brain mitochondria. J. Biol. Chem..

[86] Pitkanen S., Robinson B.H. (1996). Mitochondrial complex I deficiency leads to increased production of superoxide radicals and induction of superoxide dismutase. J. Clin. Investig..

[87] Robinson B.H. (1998). Human complex I deficiency: Clinical spectrum and involvement of oxygen free radicals in the pathogenicity of the defect. Biochim. Biophys. Acta.

[88] Carelli V., Rugolo M., Sgarbi G., Ghelli A., Zanna C., Baracca A., Lenaz G., Napoli E., Martinuzzi A., Solaini G. (2004). Bioenergetics shapes cellular death pathways in Leber’s hereditary optic neuropathy: A model of mitochondrial neurodegeneration. Biochim. Biophys. Acta.

[89] Zanna C., Ghelli A., Porcelli A.M., Carelli V., Martinuzzi A., Rugolo M. (2003). Apoptotic cell death of cybrid cells bearing Leber’s hereditary optic neuropathy mutations is caspase independent. Ann. N. Y. Acad. Sci..

[90] Yen M.Y., Wang A.G., Wei Y.H. (2006). Leber’s hereditary optic neuropathy: A multifactorial disease. Prog. Retin. Eye Res..

[91] Kirches E. (2011). LHON: Mitochondrial Mutations and More. Curr. Genom..

[92] Rovcanin B., Jancic J., Pajic J., Rovcanin M., Samardzic J., Djuric V., Nikolic B., Ivancevic N., Novakovic I., Kostic V. (2021). Oxidative Stress Profile in Genetically Confirmed Cases of Leber’s Hereditary Optic Neuropathy. J. Mol. Neurosci. MN.

[93] Delettre C., Lenaers G., Griffoin J.M., Gigarel N., Lorenzo C., Belenguer P., Pelloquin L., Grosgeorge J., Turc-Carel C., Perret E. (2000). Nuclear gene OPA1, encoding a mitochondrial dynamin-related protein, is mutated in dominant optic atrophy. Nat. Genet..

[94] Van Bergen N.J., Chakrabarti R., O’Neill E.C., Crowston J.G., Trounce I.A. (2011). Mitochondrial disorders and the eye. Eye Brain.

[95] Yu-Wai-Man P., Chinnery P.F. (2013). Dominant optic atrophy: Novel OPA1 mutations and revised prevalence estimates. Ophthalmology.

[96] Alavi M.V., Fuhrmann N. (2013). Dominant optic atrophy, OPA1, and mitochondrial quality control: Understanding mitochondrial network dynamics. Mol. Neurodegener..

[97] Frezza C., Cipolat S., Martins de Brito O., Micaroni M., Beznoussenko G.V., Rudka T., Bartoli D., Polishuck R.S., Danial N.N., De Strooper B. (2006). OPA1 controls apoptotic cristae remodeling independently from mitochondrial fusion. Cell.

[98] Sun S., Erchova I., Sengpiel F., Votruba M. (2020). Opa1 Deficiency Leads to Diminished Mitochondrial Bioenergetics With Compensatory Increased Mitochondrial Motility. Investig. Ophthalmol. Vis. Sci..

[99] Nguyen D., Alavi M.V., Kim K.Y., Kang T., Scott R.T., Noh Y.H., Lindsey J.D., Wissinger B., Ellisman M.H., Weinreb R.N. (2011). A new vicious cycle involving glutamate excitotoxicity, oxidative stress and mitochondrial dynamics. Cell Death Dis..

[100] Wong A., Cavelier L., Collins-Schramm H.E., Seldin M.F., McGrogan M., Savontaus M.L., Cortopassi G.A. (2002). Differentiation-specific effects of LHON mutations introduced into neuronal NT2 cells. Hum. Mol. Genet..

[101] Beretta S., Mattavelli L., Sala G., Tremolizzo L., Schapira A.H., Martinuzzi A., Carelli V., Ferrarese C. (2004). Leber hereditary optic neuropathy mtDNA mutations disrupt glutamate transport in cybrid cell lines. Brain A J. Neurol..

[102] Danielson S.R., Carelli V., Tan G., Martinuzzi A., Schapira A.H., Savontaus M.L., Cortopassi G.A. (2005). Isolation of transcriptomal changes attributable to LHON mutations and the cybridization process. Brain A J. Neurol..

[103] Floreani M., Napoli E., Martinuzzi A., Pantano G., De Riva V., Trevisan R., Bisetto E., Valente L., Carelli V., Dabbeni-Sala F. (2005). Antioxidant defences in cybrids harboring mtDNA mutations associated with Leber’s hereditary optic neuropathy. FEBS J..

[104] Lin C.S., Sharpley M.S., Fan W., Waymire K.G., Sadun A.A., Carelli V., Ross-Cisneros F.N., Baciu P., Sung E., McManus M.J. (2012). Mouse mtDNA mutant model of Leber hereditary optic neuropathy. Proc. Natl. Acad. Sci. USA.

[105] Biousse V., Newman N.J. (2015). Ischemic Optic Neuropathies. N. Engl. J. Med..

[106] Miller N.R., Arnold A.C. (2015). Current concepts in the diagnosis, pathogenesis and management of nonarteritic anterior ischaemic optic neuropathy. Eye.

[107] Kim J.M., Kim Y.J., Kim D.M. (2012). Increased expression of oxyproteins in the optic nerve head of an in vivo model of optic nerve ischemia. BMC Ophthalmol..

[108] Kamata H., Honda S., Maeda S., Chang L., Hirata H., Karin M. (2005). Reactive oxygen species promote TNFalpha-induced death and sustained JNK activation by inhibiting MAP kinase phosphatases. Cell.

[109] Sena L.A., Chandel N.S. (2012). Physiological roles of mitochondrial reactive oxygen species. Mol. Cell.

[110] Li W., Yang S. (2016). Targeting oxidative stress for the treatment of ischemic stroke: Upstream and downstream therapeutic strategies. Brain Circ..

[111] Soares R.O.S., Losada D.M., Jordani M.C., Évora P., Castro E.S.O. (2019). Ischemia/Reperfusion Injury Revisited: An Overview of the Latest Pharmacological Strategies. Int. J. Mol. Sci..

[112] Granger D.N., Kvietys P.R. (2015). Reperfusion injury and reactive oxygen species: The evolution of a concept. Redox Biol..

[113] Armstead W.M., Mirro R., Busija D.W., Leffler C.W. (1988). Postischemic generation of superoxide anion by newborn pig brain. Am. J. Physiol..

[114] Oka H., Kanemitsu H., Nihei H., Nakayama H., Tamura A., Sano K. (1992). Change of xanthine dehydrogenase and xanthine oxidase activities in rat brain following complete ischaemia. Neurol. Res..

[115] Kinuta Y., Kimura M., Itokawa Y., Ishikawa M., Kikuchi H. (1989). Changes in xanthine oxidase in ischemic rat brain. J. Neurosurg..

[116] Ono T., Tsuruta R., Fujita M., Aki H.S., Kutsuna S., Kawamura Y., Wakatsuki J., Aoki T., Kobayashi C., Kasaoka S. (2009). Xanthine oxidase is one of the major sources of superoxide anion radicals in blood after reperfusion in rats with forebrain ischemia/reperfusion. Brain Res..

[117] Chance B., Sies H., Boveris A. (1979). Hydroperoxide metabolism in mammalian organs. Physiol. Rev..

[118] Davies K.J., Delsignore M.E., Lin S.W. (1987). Protein damage and degradation by oxygen radicals. II. Modification of amino acids. J. Biol. Chem..

[119] Flammer J., Mozaffarieh M. (2007). What is the present pathogenetic concept of glaucomatous optic neuropathy?. Surv. Ophthalmol..

[120] Kang J.S., Tian J.H., Pan P.Y., Zald P., Li C., Deng C., Sheng Z.H. (2008). Docking of axonal mitochondria by syntaphilin controls their mobility and affects short-term facilitation. Cell.

[121] Errea O., Moreno B., Gonzalez-Franquesa A., Garcia-Roves P.M., Villoslada P. (2015). The disruption of mitochondrial axonal transport is an early event in neuroinflammation. J. Neuroinflamm..

[122] Zheng Y., Zhang X., Wu X., Jiang L., Ahsan A., Ma S., Xiao Z., Han F., Qin Z.H., Hu W. (2019). Somatic autophagy of axonal mitochondria in ischemic neurons. J. Cell Biol..

[123] Zhang C., Guo Y., Miller N.R., Bernstein S.L. (2009). Optic nerve infarction and post-ischemic inflammation in the rodent model of anterior ischemic optic neuropathy (rAION). Brain Res..

[124] Bernardo-Colón A., Vest V., Clark A., Cooper M.L., Calkins D.J., Harrison F.E., Rex T.S. (2018). Antioxidants prevent inflammation and preserve the optic projection and visual function in experimental neurotrauma. Cell Death Dis..

[125] Steinsapir K.D., Goldberg R.A. (1994). Traumatic optic neuropathy. Surv. Ophthalmol..

[126] Steinsapir K.D. (1999). Traumatic optic neuropathy. Curr. Opin. Ophthalmol..

[127] Li H.Y., Ruan Y.W., Ren C.R., Cui Q., So K.F. (2014). Mechanisms of secondary degeneration after partial optic nerve transection. Neural Regen. Res..

[128] Farkas O., Povlishock J.T. (2007). Cellular and subcellular change evoked by diffuse traumatic brain injury: A complex web of change extending far beyond focal damage. Prog. Brain Res..

[129] Bastakis G.G., Ktena N., Karagogeos D., Savvaki M. (2019). Models and treatments for traumatic optic neuropathy and demyelinating optic neuritis. Dev. Neurobiol..

[130] Cansler S.M., Evanson N.K. (2020). Connecting endoplasmic reticulum and oxidative stress to retinal degeneration, TBI, and traumatic optic neuropathy. J. Neurosci. Res..

[131] Fitzgerald M., Bartlett C.A., Harvey A.R., Dunlop S.A. (2010). Early events of secondary degeneration after partial optic nerve transection: An immunohistochemical study. J. Neurotrauma.

[132] Wells J., Kilburn M.R., Shaw J.A., Bartlett C.A., Harvey A.R., Dunlop S.A., Fitzgerald M. (2012). Early in vivo changes in calcium ions, oxidative stress markers, and ion channel immunoreactivity following partial injury to the optic nerve. J. Neurosci. Res..

[133] Ahmad S., Fatteh N., El-Sherbiny N.M., Naime M., Ibrahim A.S., El-Sherbini A.M., El-Shafey S.A., Khan S., Fulzele S., Gonzales J. (2013). Potential role of A2A adenosine receptor in traumatic optic neuropathy. J. Neuroimmunol.

[134] Peng T.I., Jou M.J. (2010). Oxidative stress caused by mitochondrial calcium overload. Ann. N. Y. Acad. Sci..

[135] Payne S.C., Bartlett C.A., Harvey A.R., Dunlop S.A., Fitzgerald M. (2012). Myelin sheath decompaction, axon swelling, and functional loss during chronic secondary degeneration in rat optic nerve. Investig. Ophthalmol. Vis. Sci..

[136] Park E., Velumian A.A., Fehlings M.G. (2004). The role of excitotoxicity in secondary mechanisms of spinal cord injury: A review with an emphasis on the implications for white matter degeneration. J. Neurotrauma.

[137] Szymanski C.R., Chiha W., Morellini N., Cummins N., Bartlett C.A., O’Hare Doig R.L., Savigni D.L., Payne S.C., Harvey A.R., Dunlop S.A. (2013). Paranode Abnormalities and Oxidative Stress in Optic Nerve Vulnerable to Secondary Degeneration: Modulation by 670 nm Light Treatment. PLoS ONE.

[138] O’Hare Doig R.L., Bartlett C.A., Maghzal G.J., Lam M., Archer M., Stocker R., Fitzgerald M. (2014). Reactive species and oxidative stress in optic nerve vulnerable to secondary degeneration. Exp. Neurol..

[139] Cummins N., Bartlett C.A., Archer M., Bartlett E., Hemmi J.M., Harvey A.R., Dunlop S.A., Fitzgerald M. (2013). Changes to mitochondrial ultrastructure in optic nerve vulnerable to secondary degeneration in vivo are limited by irradiation at 670 nm. BMC NeuroSci..

[140] Levkovitch-Verbin H., Harris-Cerruti C., Groner Y., Wheeler L.A., Schwartz M., Yoles E. (2000). RGC death in mice after optic nerve crush injury: Oxidative stress and neuroprotection. Investig. Ophthalmol. Vis. Sci..

[141] Lieven C.J., Hoegger M.J., Schlieve C.R., Levin L.A. (2006). Retinal ganglion cell axotomy induces an increase in intracellular superoxide anion. Investig. Ophthalmol. Vis. Sci..

[142] Hoorbakht H., Bagherkashi F. (2012). Optic neuritis, its differential diagnosis and management. Open OphthalMol. J..

[143] Toosy A.T., Mason D.F., Miller D.H. (2014). Optic neuritis. Lancet Neurol..

[144] Youl B.D., Turano G., Miller D.H., Towell A.D., MacManus D.G., Moore S.G., Jones S.J., Barrett G., Kendall B.E., Moseley I.F. (1991). The pathophysiology of acute optic neuritis. An association of gadolinium leakage with clinical and electrophysiological deficits. Brain A J. Neurol..

[145] Pau D., Al Zubidi N., Yalamanchili S., Plant G.T., Lee A.G. (2011). Optic neuritis. Eye.

[146] Biswas S.K. (2016). Does the Interdependence between Oxidative Stress and Inflammation Explain the Antioxidant Paradox?. Oxidative Med. Cell. Longev..

[147] Shu Y., Li R., Qiu W., Chang Y., Sun X., Fang L., Chen C., Yang Y., Lu Z., Hu X. (2017). Association of serum gamma-glutamyltransferase and C-reactive proteins with neuromyelitis optica and multiple sclerosis. Mult. Scler. Relat. Disord..

[148] Falardeau J., Fryman A., Wanchu R., Marracci G.H., Mass M., Wooliscroft L., Bourdette D.N., Murchison C.F., Hills W.L., Yadav V. (2019). Oral lipoic acid as a treatment for acute optic neuritis: A blinded, placebo controlled randomized trial. Mult. Scler. J. Exp. Transl. Clin..

[149] Guy J., Ellis E.A., Hope G.M., Rao N.A. (1986). Influence of antioxidant enzymes in reduction of optic disc edema in experimental optic neuritis. J. Free Radic. Biol. Med..

[150] Kimura A., Namekata K., Guo X., Noro T., Harada C., Harada T. (2017). Targeting Oxidative Stress for Treatment of Glaucoma and Optic Neuritis. Oxidative Med. Cell. Longev..

[151] Khan R.S., Fonseca-Kelly Z., Callinan C., Zuo L., Sachdeva M.M., Shindler K.S. (2012). SIRT1 activating compounds reduce oxidative stress and prevent cell death in neuronal cells. Front. Cell NeuroSci..

[152] Guy J., Ellis E.A., Hope G.M., Rao N.A. (1989). Antioxidant enzyme suppression of demyelination in experimental optic neuritis. Curr. Eye Res..

[153] Guy J., Ellis E.A., Rao N.A. (1990). Hydrogen peroxide localization in experimental optic neuritis. Arch. Ophthalmol..

[154] Qi X., Lewin A.S., Sun L., Hauswirth W.W., Guy J. (2007). Suppression of mitochondrial oxidative stress provides long-term neuroprotection in experimental optic neuritis. Investig. Ophthalmol. Vis. Sci..

[155] Qi X., Sun L., Lewin A.S., Hauswirth W.W., Guy J. (2007). Long-term suppression of neurodegeneration in chronic experimental optic neuritis: Antioxidant gene therapy. Investig. Ophthalmol. Vis. Sci..

[156] Larabee C.M., Desai S., Agasing A., Georgescu C., Wren J.D., Axtell R.C., Plafker S.M. (2016). Loss of Nrf2 exacerbates the visual deficits and optic neuritis elicited by experimental autoimmune encephalomyelitis. Mol. Vis..

[157] Zhang J., Fang F., Li L., Huang H., Webber H.C., Sun Y., Mahajan V.B., Hu Y. (2019). A Reversible Silicon Oil-Induced Ocular Hypertension Model in Mice. J. Vis. Exp..

[158] Conti F., Romano G.L., Eandi C.M., Toro M.D., Rejdak R., Di Benedetto G., Lazzara F., Bernardini R., Drago F., Cantarella G. (2021). Brimonidine is Neuroprotective in Animal Paradigm of Retinal Ganglion Cell Damage. Front. Pharmacol..

[159] Benozzi J., Nahum L.P., Campanelli J.L., Rosenstein R.E. (2002). Effect of hyaluronic acid on intraocular pressure in rats. Investig. Ophthalmol. Vis. Sci..

[160] Ueda J., Sawaguchi S., Hanyu T., Yaoeda K., Fukuchi T., Abe H., Ozawa H. (1998). Experimental glaucoma model in the rat induced by laser trabecular photocoagulation after an intracameral injection of India ink. Jpn. J. Ophthalmol..

[161] Grozdanic S.D., Betts D.M., Sakaguchi D.S., Allbaugh R.A., Kwon Y.H., Kardon R.H. (2003). Laser-induced mouse model of chronic ocular hypertension. Investig. Ophthalmol. Vis. Sci..

[162] Ji J., Chang P., Pennesi M.E., Yang Z., Zhang J., Li D., Wu S.M., Gross R.L. (2005). Effects of elevated intraocular pressure on mouse retinal ganglion cells. Vis. Res..

[163] Ruiz-Ederra J., Verkman A.S. (2006). Mouse model of sustained elevation in intraocular pressure produced by episcleral vein occlusion. Exp. Eye Res..

[164] Sheldon W.G., Warbritton A.R., Bucci T.J., Turturro A. (1995). Glaucoma in food-restricted and ad libitum-fed DBA/2NNia mice. Lab. Anim. Sci..

[165] Libby R.T., Anderson M.G., Pang I.H., Robinson Z.H., Savinova O.V., Cosma I.M., Snow A., Wilson L.A., Smith R.S., Clark A.F. (2005). Inherited glaucoma in DBA/2J mice: Pertinent disease features for studying the neurodegeneration. Vis. NeuroSci..

[166] Chang B., Smith R.S., Hawes N.L., Anderson M.G., Zabaleta A., Savinova O., Roderick T.H., Heckenlively J.R., Davisson M.T., John S.W. (1999). Interacting loci cause severe iris atrophy and glaucoma in DBA/2J mice. Nat. Genet..

[167] John S.W. (2005). Mechanistic insights into glaucoma provided by experimental genetics the cogan lecture. Investig. Ophthalmol. Vis. Sci..

[168] Wilkins H.M., Carl S.M., Swerdlow R.H. (2014). Cytoplasmic hybrid (cybrid) cell lines as a practical model for mitochondriopathies. Redox Biol..

[169] Jun A.S., Brown M.D., Wallace D.C. (1994). A mitochondrial DNA mutation at nucleotide pair 14459 of the NADH dehydrogenase subunit 6 gene associated with maternally inherited Leber hereditary optic neuropathy and dystonia. Proc. Natl. Acad. Sci. USA.

[170] Malfatti E., Bugiani M., Invernizzi F., de Souza C.F., Farina L., Carrara F., Lamantea E., Antozzi C., Confalonieri P., Sanseverino M.T. (2007). Novel mutations of ND genes in complex I deficiency associated with mitochondrial encephalopathy. Brain A J. Neurol..

[171] Alavi M.V., Bette S., Schimpf S., Schuettauf F., Schraermeyer U., Wehrl H.F., Ruttiger L., Beck S.C., Tonagel F., Pichler B.J. (2007). A splice site mutation in the murine Opa1 gene features pathology of autosomal dominant optic atrophy. Brain A J. Neurol..

[172] Davies V.J., Hollins A.J., Piechota M.J., Yip W., Davies J.R., White K.E., Nicols P.P., Boulton M.E., Votruba M. (2007). Opa1 deficiency in a mouse model of autosomal dominant optic atrophy impairs mitochondrial morphology, optic nerve structure and visual function. Hum. Mol. Genet..

[173] Chang C.H., Huang T.L., Huang S.P., Tsai R.K. (2014). Neuroprotective effects of recombinant human granulocyte colony-stimulating factor (G-CSF) in a rat model of anterior ischemic optic neuropathy (rAION). Exp. Eye Res..

[174] Wen Y.T., Huang T.L., Huang S.P., Chang C.H., Tsai R.K. (2016). Early applications of granulocyte colony-stimulating factor (G-CSF) can stabilize the blood-optic-nerve barrier and ameliorate inflammation in a rat model of anterior ischemic optic neuropathy (rAION). Dis. Model. Mech..

[175] Lin W.N., Kapupara K., Wen Y.T., Chen Y.H., Pan I.H., Tsai R.K. (2020). Haematococcus pluvialis-Derived Astaxanthin Is a Potential Neuroprotective Agent against Optic Nerve Ischemia. Mar. Drugs.

[176] Liu P.K., Wen Y.T., Lin W., Kapupara K., Tai M., Tsai R.K. (2020). Neuroprotective effects of low-dose G-CSF plus meloxicam in a rat model of anterior ischemic optic neuropathy. Sci. Rep..

[177] Bernstein S.L., Guo Y., Kelman S.E., Flower R.W., Johnson M.A. (2003). Functional and cellular responses in a novel rodent model of anterior ischemic optic neuropathy. Investig. Ophthalmol. Vis. Sci..

[178] Slater B.J., Mehrabian Z., Guo Y., Hunter A., Bernstein S.L. (2008). Rodent anterior ischemic optic neuropathy (rAION) induces regional retinal ganglion cell apoptosis with a unique temporal pattern. Investig. Ophthalmol. Vis. Sci..

[179] Bernardo-Colón A., Vest V., Cooper M.L., Naguib S.A., Calkins D.J., Rex T.S. (2019). Progression and Pathology of Traumatic Optic Neuropathy From Repeated Primary Blast Exposure. Front. NeuroSci..

[180] DeJulius C.R., Bernardo-Colón A., Naguib S., Backstrom J.R., Kavanaugh T., Gupta M.K., Duvall C.L., Rex T.S. (2021). Microsphere antioxidant and sustained erythropoietin-R76E release functions cooperate to reduce traumatic optic neuropathy. J. Control Release.

[181] Hines-Beard J., Marchetta J., Gordon S., Chaum E., Geisert E.E., Rex T.S. (2012). A mouse model of ocular blast injury that induces closed globe anterior and posterior pole damage. Exp. Eye Res..

[182] Tang Z., Zhang S., Lee C., Kumar A., Arjunan P., Li Y., Zhang F., Li X. (2011). An optic nerve crush injury murine model to study retinal ganglion cell survival. J. Vis. Exp..

[183] Huang S.P., Fang K.T., Chang C.H., Huang T.L., Wen Y.T., Tsai R.K. (2016). Autocrine protective mechanisms of human granulocyte colony-stimulating factor (G-CSF) on retinal ganglion cells after optic nerve crush. Exp. Eye Res..

[184] Huang Y., Xu Y., Cheng Q., Yu S., Gao Y., Shu Q., Yang C., Sun Y., Wang J., Xu F. (2014). The expression changes of myelin and lymphocyte protein (MAL) following optic nerve crush in adult rats retinal ganglion cells. J. Mol. Neurosci. MN.

[185] Shi Y., Ye D., Huang R., Xu Y., Lu P., Chen H., Huang J. (2020). Down Syndrome Critical Region 1 Reduces Oxidative Stress-Induced Retinal Ganglion Cells Apoptosis via CREB-Bcl-2 Pathway. Investig. Ophthalmol. Vis. Sci..

[186] Sarikcioglu L., Demir N., Demirtop A. (2007). A standardized method to create optic nerve crush: Yasargil aneurysm clip. Exp. Eye Res..

[187] Tsai R.K., Chang C.H., Wang H.Z. (2008). Neuroprotective effects of recombinant human granulocyte colony-stimulating factor (G-CSF) in neurodegeneration after optic nerve crush in rats. Exp. Eye Res..

[188] Ye D., Shi Y., Xu Y., Huang J. (2019). PACAP Attenuates Optic Nerve Crush-Induced Retinal Ganglion Cell Apoptosis Via Activation of the CREB-Bcl-2 Pathway. J. Mol. Neurosci. MN.

[189] Vest V., Bernardo-Colón A., Watkins D., Kim B., Rex T.S. (2019). Rapid Repeat Exposure to Subthreshold Trauma Causes Synergistic Axonal Damage and Functional Deficits in the Visual Pathway in a Mouse Model. J. Neurotrauma.

[190] Bricker-Anthony C., Hines-Beard J., Rex T.S. (2014). Molecular changes and vision loss in a mouse model of closed-globe blast trauma. Investig. Ophthalmol. Vis. Sci..

[191] Cockerham G.C., Rice T.A., Hewes E.H., Cockerham K.P., Lemke S., Wang G., Lin R.C., Glynn-Milley C., Zumhagen L. (2011). Closed-eye ocular injuries in the Iraq and Afghanistan wars. N. Engl. J. Med..

[192] Chaudhary P., Marracci G., Yu X., Galipeau D., Morris B., Bourdette D. (2011). Lipoic acid decreases inflammation and confers neuroprotection in experimental autoimmune optic neuritis. J. Neuroimmunol.

[193] Dietrich M., Helling N., Hilla A., Heskamp A., Issberner A., Hildebrandt T., Kohne Z., Küry P., Berndt C., Aktas O. (2018). Early alpha-lipoic acid therapy protects from degeneration of the inner retinal layers and vision loss in an experimental autoimmune encephalomyelitis-optic neuritis model. J. Neuroinflamm..

[194] Constantinescu C.S., Farooqi N., O’Brien K., Gran B. (2011). Experimental autoimmune encephalomyelitis (EAE) as a model for multiple sclerosis (MS). Br. J. Pharm..

[195] Wisniewski H.M., Madrid R.E. (1983). Chronic progressive experimental allergic encephalomyelitis (EAE) in adult guinea pigs. J. Neuropathol Exp. Neurol..

[196] Aranda M.L., Dorfman D., Sande P.H., Rosenstein R.E. (2015). Experimental optic neuritis induced by the microinjection of lipopolysaccharide into the optic nerve. Exp. Neurol..

[197] Aranda M.L., González Fleitas M.F., De Laurentiis A., Keller Sarmiento M.I., Chianelli M., Sande P.H., Dorfman D., Rosenstein R.E. (2016). Neuroprotective effect of melatonin in experimental optic neuritis in rats. J. Pineal Res..

[198] Tanaka T., Yokoi T., Tamalu F., Watanabe S., Nishina S., Azuma N. (2015). Generation of retinal ganglion cells with functional axons from human induced pluripotent stem cells. Sci. Rep..

[199] Ohlemacher S.K., Sridhar A., Xiao Y., Hochstetler A.E., Sarfarazi M., Cummins T.R., Meyer J.S. (2016). Stepwise Differentiation of Retinal Ganglion Cells from Human Pluripotent Stem Cells Enables Analysis of Glaucomatous Neurodegeneration. Stem Cells.

[200] Fligor C.M., Langer K.B., Sridhar A., Ren Y., Shields P.K., Edler M.C., Ohlemacher S.K., Sluch V.M., Zack D.J., Zhang C. (2018). Three-Dimensional Retinal Organoids Facilitate the Investigation of Retinal Ganglion Cell Development, Organization and Neurite Outgrowth from Human Pluripotent Stem Cells. Sci. Rep..

[201] Teotia P., Chopra D.A., Dravid S.M., Van Hook M.J., Qiu F., Morrison J., Rizzino A., Ahmad I. (2017). Generation of Functional Human Retinal Ganglion Cells with Target Specificity from Pluripotent Stem Cells by Chemically Defined Recapitulation of Developmental Mechanism. Stem Cells.

[202] Chavali V.R.M., Haider N., Rathi S., Vrathasha V., Alapati T., He J., Gill K., Nikonov R., Duong T.T., McDougald D.S. (2020). Dual SMAD inhibition and Wnt inhibition enable efficient and reproducible differentiations of induced pluripotent stem cells into retinal ganglion cells. Sci. Rep..

[203] Langer K.B., Ohlemacher S.K., Phillips M.J., Fligor C.M., Jiang P., Gamm D.M., Meyer J.S. (2018). Retinal Ganglion Cell Diversity and Subtype Specification from Human Pluripotent Stem Cells. Stem Cell Rep..

[204] VanderWall K.B., Vij R., Ohlemacher S.K., Sridhar A., Fligor C.M., Feder E.M., Edler M.C., Baucum A.J., Cummins T.R., Meyer J.S. (2019). Astrocytes Regulate the Development and Maturation of Retinal Ganglion Cells Derived from Human Pluripotent Stem Cells. Stem Cell Rep..

[205] Zhong X., Gutierrez C., Xue T., Hampton C., Vergara M.N., Cao L.H., Peters A., Park T.S., Zambidis E.T., Meyer J.S. (2014). Generation of three-dimensional retinal tissue with functional photoreceptors from human iPSCs. Nat. Commun..

[206] Manafi N., Shokri F., Achberger K., Hirayama M., Mohammadi M.H., Noorizadeh F., Hong J., Liebau S., Tsuji T., Quinn P.M.J. (2021). Organoids and organ chips in ophthalmology. Ocul. Surf..

[207] Fligor C.M., Lavekar S.S., Harkin J., Shields P.K., VanderWall K.B., Huang K.C., Gomes C., Meyer J.S. (2021). Extension of retinofugal projections in an assembled model of human pluripotent stem cell-derived organoids. Stem Cell Rep..

[208] Wagstaff P.E., Ten Asbroek A., Ten Brink J.B., Jansonius N.M., Bergen A.A.B. (2021). An alternative approach to produce versatile retinal organoids with accelerated ganglion cell development. Sci. Rep..

[209] Tucker B.A., Solivan-Timpe F., Roos B.R., Anfinson K.R., Robin A.L., Wiley L.A., Mullins R.F., Fingert J.H. (2014). Duplication of TBK1 Stimulates Autophagy in iPSC-derived Retinal Cells from a Patient with Normal Tension Glaucoma. J. Stem Cell Res..

[210] Teotia P., Van Hook M.J., Wichman C.S., Allingham R.R., Hauser M.A., Ahmad I. (2017). Modeling Glaucoma: Retinal Ganglion Cells Generated from Induced Pluripotent Stem Cells of Patients with SIX6 Risk Allele Show Developmental Abnormalities. Stem Cells.

[211] Wong R.C.B., Lim S.Y., Hung S.S.C., Jackson S., Khan S., Van Bergen N.J., De Smit E., Liang H.H., Kearns L.S., Clarke L. (2017). Mitochondrial replacement in an iPSC model of Leber’s hereditary optic neuropathy. Aging.

[212] Wu Y.R., Wang A.G., Chen Y.T., Yarmishyn A.A., Buddhakosai W., Yang T.C., Hwang D.K., Yang Y.P., Shen C.N., Lee H.C. (2018). Bioactivity and gene expression profiles of hiPSC-generated retinal ganglion cells in MT-ND4 mutated Leber’s hereditary optic neuropathy. Exp. Cell Res..

[213] Yang T.C., Yarmishyn A.A., Yang Y.P., Lu P.C., Chou S.J., Wang M.L., Lin T.C., Hwang D.K., Chou Y.B., Chen S.J. (2020). Mitochondrial transport mediates survival of retinal ganglion cells in affected LHON patients. Hum. Mol. Genet..

[214] Yang Y.P., Nguyen P.N.N., Lin T.C., Yarmishyn A.A., Chen W.S., Hwang D.K., Chiou G.Y., Lin T.W., Chien C.S., Tsai C.Y. (2019). Glutamate Stimulation Dysregulates AMPA Receptors-Induced Signal Transduction Pathway in Leber’s Inherited Optic Neuropathy Patient-Specific hiPSC-Derived Retinal Ganglion Cells. Cells.

[215] Oh-ishi S., Hayashi I., Hayashi M., Yamaki K., Utsunomiya I. (1989). Pharmacological demonstration of inflammatory mediators using experimental inflammatory models: Rat pleurisy induced by carrageenin and phorbol myristate acetate. Dermatologica.

[216] Galera-Monge T., Zurita-Diaz F., Moreno-Izquierdo A., Fraga M.F., Fernandez A.F., Ayuso C., Garesse R., Gallardo M.E. (2016). Generation of a human iPSC line from a patient with an optic atrophy ‘plus’ phenotype due to a mutation in the OPA1 gene. Stem Cell Res..

[217] Zurita-Diaz F., Galera-Monge T., Moreno-Izquierdo A., Corton M., Ayuso C., Garesse R., Gallardo M.E. (2017). Establishment of a human DOA ‘plus’ iPSC line, IISHDOi003-A, with the mutation in the OPA1 gene: C.1635C>A.; p.Ser545Arg. Stem Cell Res..

[218] Sladen P.E., Perdigao P.R.L., Salsbury G., Novoselova T., van der Spuy J., Chapple J.P., Yu-Wai-Man P., Cheetham M.E. (2021). CRISPR-Cas9 correction of OPA1 c.1334G>A: P.R445H restores mitochondrial homeostasis in dominant optic atrophy patient-derived iPSCs. Mol. Nucleic Acids.

[219] Quinn P.M.J., Moreira P.I., Ambrosio A.F., Alves C.H. (2020). PINK1/PARKIN signalling in neurodegeneration and neuroinflammation. Acta Neuropathol. Commun..

[220] Inagaki S., Kawase K., Funato M., Seki J., Kawase C., Ohuchi K., Kameyama T., Ando S., Sato A., Morozumi W. (2018). Effect of Timolol on Optineurin Aggregation in Transformed Induced Pluripotent Stem Cells Derived From Patient with Familial Glaucoma. Investig. OphthalMol. Vis. Sci..

[221] VanderWall K.B., Huang K.C., Pan Y., Lavekar S.S., Fligor C.M., Allsop A.R., Lentsch K.A., Dang P., Zhang C., Tseng H.C. (2020). Retinal Ganglion Cells With a Glaucoma OPTN(E50K) Mutation Exhibit Neurodegenerative Phenotypes when Derived from Three-Dimensional Retinal Organoids. Stem Cell Rep..

[222] Lee D., Shim M.S., Kim K.Y., Noh Y.H., Kim H., Kim S.Y., Weinreb R.N., Ju W.K. (2014). Coenzyme Q10 inhibits glutamate excitotoxicity and oxidative stress-mediated mitochondrial alteration in a mouse model of glaucoma. Investig. Ophthalmol. Vis. Sci..

[223] Nucci C., Tartaglione R., Cerulli A., Mancino R., Spanò A., Cavaliere F., Rombolà L., Bagetta G., Corasaniti M.T., Morrone L.A. (2007). Retinal damage caused by high intraocular pressure-induced transient ischemia is prevented by coenzyme Q10 in rat. Int. Rev. Neurobiol..

[224] Ozdemir G., Tolun F.I., Gul M., Imrek S. (2009). Retinal oxidative stress induced by intraocular hypertension in rats may be ameliorated by brimonidine treatment and N-acetyl cysteine supplementation. J. Glaucoma.

[225] Inman D.M., Lambert W.S., Calkins D.J., Horner P.J. (2013). α-Lipoic acid antioxidant treatment limits glaucoma-related retinal ganglion cell death and dysfunction. PLoS ONE.

[226] Chou T.H., Romano G.L., Amato R., Porciatti V. (2020). Nicotinamide-Rich Diet in DBA/2J Mice Preserves Retinal Ganglion Cell Metabolic Function as Assessed by PERG Adaptation to Flicker. Nutrients.

[227] Yamamoto K., Maruyama K., Himori N., Omodaka K., Yokoyama Y., Shiga Y., Morin R., Nakazawa T. (2014). The novel Rho kinase (ROCK) inhibitor K-115: A new candidate drug for neuroprotective treatment in glaucoma. Investig. Ophthalmol. Vis. Sci..

[228] Akaiwa K., Namekata K., Azuchi Y., Guo X., Kimura A., Harada C., Mitamura Y., Harada T. (2017). Edaravone suppresses retinal ganglion cell death in a mouse model of normal tension glaucoma. Cell Death Dis..

[229] Haroon M.F., Fatima A., Schöler S., Gieseler A., Horn T.F., Kirches E., Wolf G., Kreutzmann P. (2007). Minocycline, a possible neuroprotective agent in Leber’s hereditary optic neuropathy (LHON): Studies of cybrid cells bearing 11,778 mutation. Neurobiol. Dis..

[230] Ghelli A., Porcelli A.M., Zanna C., Martinuzzi A., Carelli V., Rugolo M. (2008). Protection against oxidant-induced apoptosis by exogenous glutathione in Leber hereditary optic neuropathy cybrids. Investig. Ophthalmol. Vis. Sci..

[231] Sadun A.A., Chicani C.F., Ross-Cisneros F.N., Barboni P., Thoolen M., Shrader W.D., Kubis K., Carelli V., Miller G. (2012). Effect of EPI-743 on the clinical course of the mitochondrial disease Leber hereditary optic neuropathy. Arch Neurol..

[232] Klopstock T., Yu-Wai-Man P., Dimitriadis K., Rouleau J., Heck S., Bailie M., Atawan A., Chattopadhyay S., Schubert M., Garip A. (2011). A randomized placebo-controlled trial of idebenone in Leber’s hereditary optic neuropathy. Brain A J. Neurol..

[233] Carelli V., La Morgia C., Valentino M.L., Rizzo G., Carbonelli M., De Negri A.M., Sadun F., Carta A., Guerriero S., Simonelli F. (2011). Idebenone treatment in Leber’s hereditary optic neuropathy. Brain A J. Neurol..

[234] Klopstock T., Metz G., Yu-Wai-Man P., Büchner B., Gallenmüller C., Bailie M., Nwali N., Griffiths P.G., von Livonius B., Reznicek L. (2013). Persistence of the treatment effect of idebenone in Leber’s hereditary optic neuropathy. Brain A J. Neurol..

[235] Barboni P., Valentino M.L., La Morgia C., Carbonelli M., Savini G., De Negri A., Simonelli F., Sadun F., Caporali L., Maresca A. (2013). Idebenone treatment in patients with OPA1-mutant dominant optic atrophy. Brain A J. Neurol..

[236] Romagnoli M., La Morgia C., Carbonelli M., Di Vito L., Amore G., Zenesini C., Cascavilla M.L., Barboni P., Carelli V. (2020). Idebenone increases chance of stabilization/recovery of visual acuity in OPA1-dominant optic atrophy. Ann. Clin. Transl. Neurol..

[237] Goldenberg-Cohen N., Dadon-Bar-El S., Hasanreisoglu M., Avraham-Lubin B.C., Dratviman-Storobinsky O., Cohen Y., Weinberger D. (2009). Possible neuroprotective effect of brimonidine in a mouse model of ischaemic optic neuropathy. Clin. Exp. Ophthalmol..

[238] Wen Y.T., Huang C.W., Liu C.P., Chen C.H., Tu C.M., Hwang C.S., Chen Y.H., Chen W.R., Lin K.L., Ho Y.C. (2021). Inhibition of Retinal Ganglion Cell Loss By a Novel ROCK Inhibitor (E212) in Ischemic Optic Nerve Injury Via Antioxidative and Anti-Inflammatory Actions. Investig. Ophthalmol. Vis. Sci..

[239] Sanjari N., Pakravan M., Nourinia R., Esfandiari H., Hafezi-Moghadam A., Zandi S., Nakao S., Shah-Heidari M.H., Jamali A., Yaseri M. (2016). Intravitreal Injection of a Rho-Kinase Inhibitor (Fasudil) for Recent-Onset Nonarteritic Anterior Ischemic Optic Neuropathy. J. Clin. Pharm..

[240] Fitzgerald M., Bartlett C.A., Evill L., Rodger J., Harvey A.R., Dunlop S.A. (2009). Secondary degeneration of the optic nerve following partial transection: The benefits of lomerizine. Exp. Neurol..

[241] Naguib S., Bernardo-Colón A., Cencer C., Gandra N., Rex T.S. (2020). Galantamine protects against synaptic, axonal, and vision deficits in experimental neurotrauma. Neurobiol. Dis..

[242] Cho H.K., Kim S., Lee E.J., Kee C. (2019). Neuroprotective Effect of Ginkgo Biloba Extract Against Hypoxic Retinal Ganglion Cell Degeneration In Vitro and In Vivo. J. Med. Food.

[243] Kang T.K., Le T.T., Kim K.A., Kim Y.J., Lee W.B., Jung S.H. (2021). Roots of Lithospermum erythrorhizon promotes retinal cell survival in optic nerve crush-induced retinal degeneration. Exp. Eye Res..

[244] Gaydar V., Ezrachi D., Dratviman-Storobinsky O., Hofstetter S., Avraham-Lubin B.C., Goldenberg-Cohen N. (2011). Reduction of apoptosis in ischemic retinas of two mouse models using hyperbaric oxygen treatment. Investig. Ophthalmol. Vis. Sci..

[245] Zhang H.K., Ye Y., Li K.J., Zhao Z.N., He J.F. (2020). Gypenosides Prevent H(2)O(2)-Induced Retinal Ganglion Cell Apoptosis by Concurrently Suppressing the Neuronal Oxidative Stress and Inflammatory Response. J. Mol. Neurosci. MN.

[246] Guo X., Harada C., Namekata K., Kimura A., Mitamura Y., Yoshida H., Matsumoto Y., Harada T. (2011). Spermidine alleviates severity of murine experimental autoimmune encephalomyelitis. Investig. Ophthalmol. Vis. Sci..

[247] Papucci L., Schiavone N., Witort E., Donnini M., Lapucci A., Tempestini A., Formigli L., Zecchi-Orlandini S., Orlandini G., Carella G. (2003). Coenzyme q10 prevents apoptosis by inhibiting mitochondrial depolarization independently of its free radical scavenging property. J. Biol. Chem..

[248] Sena D.F., Lindsley K. (2017). Neuroprotection for treatment of glaucoma in adults. Cochrane Database Syst. Rev..

[249] Abbhi V., Saini L., Mishra S., Sethi G., Kumar A.P., Piplani P. (2017). Design and synthesis of benzimidazole-based Rho kinase inhibitors for the treatment of glaucoma. Bioorg. Med. Chem..

[250] Hoy S.M. (2018). Netarsudil Ophthalmic Solution 0.02%: First Global Approval. Drugs.

[251] Amore G., Romagnoli M., Carbonelli M., Barboni P., Carelli V., La Morgia C. (2021). Therapeutic Options in Hereditary Optic Neuropathies. Drugs.

[252] Theodorou-Kanakari A., Karampitianis S., Karageorgou V., Kampourelli E., Kapasakis E., Theodossiadis P., Chatziralli I. (2018). Current and Emerging Treatment Modalities for Leber’s Hereditary Optic Neuropathy: A Review of the Literature. Adv. Ther..

[253] Lyseng-Williamson K.A. (2016). Idebenone: A Review in Leber’s Hereditary Optic Neuropathy. Drugs.

[254] Benedetti S., Lamorgese A., Piersantelli M., Pagliarani S., Benvenuti F., Canestrari F. (2004). Oxidative stress and antioxidant status in patients undergoing prolonged exposure to hyperbaric oxygen. Clin. Biochem..

